# Synthesis of novel pyrazolone candidates with studying some biological activities and in-silico studies

**DOI:** 10.1038/s41598-023-43575-z

**Published:** 2023-11-06

**Authors:** Magda H. Abdellattif, Eman O. Hamed, Nourhan Kh. R. Elhoseni, Mohamed G. Assy, Abdul-Hamid M. Emwas, Mariusz Jaremko, Ismail Celik, Abderrahim Titi, Krishna Kumar Yadav, Marwa S. Elgendy, Wesam S. Shehab

**Affiliations:** 1 Department of Chemistry, Sciences College, Taif University, P. O. Box 11099, 21944 Taif, Saudi Arabia; 2 Department of Chemistry, Faculty of Science, Zagazig University, Zagazig, 44519 Egypt; 3 Core Labs, King Abdullah University of Science and Technology (KAUST), 23955-6900 Thuwal, Saudi Arabia; 4 Biological and Environmental Science and Engineering (BESE), King Abdullah University of Science and Technology (KAUST), 23955-6900 Thuwal, Saudi Arabia; 5 Department of Pharmaceutical Chemistry, Faculty of Pharmacy, Erciyes University, Kayseri, 38039 Turkey; 6 Laboratory of Applied and Environmental Chemistry (LCAE), Mohamed First University, Oujda, Morocco; 7 Faculty of Science and Technology, Madhyanchal Professional University, Ratibad, Bhopal, 462044 India; 8 Department of Civil and Environmental Engineering, Faculty of Engineering, PSU Energy Systems Research Institute, Prince of Songkla University, Hat Yai, Songkhla, 90110 Thailand; 9 Department of Chemistry, Faculty of Sciences, Alazhar University (Girls), Cairo, Egypt

**Keywords:** Biochemistry, Drug discovery, Chemistry

## Abstract

Pyranopyrazole derivatives have a vital role in the class of organic compounds because of their broad spectrum of biological and pharmacological importance. Our current goal is the **[3 + 3]** cycloaddition of benzoyl isothiocyanate and pyrazolone **1** to undergo oxidation cyclization, producing pyrazoloxadiazine **3**. The diol **5** was obtained as a condensation of two equivalents of **1** with thiophene-2-carboxaldehyde in acetic acid above the sodium acetate mixture. When the condensation was carried out in piperidine under fusion, unsaturated ketone **4** was obtained. The pyrazolo pyran derivative **11** resulted from the **[3 + 3]** cycloaddition of **1** and cinnamic acid, while the Pyrone derivative was prepared by acylation of **12** with two equivalents of acetic anhydride. Phthalic anhydride undergoes arylation using zinc chloride as a catalyst. The cyclic keto acid **23** was synthesized by the action of succinic anhydride on **12** in the acetic medium, while the latter reacted with cinnamic acid, leading to pyrazole derivative **24**. All of these reactions were through the Michael reaction mechanism. All the tested compounds showed good antimicrobial activity against pathogenic microorganisms; newly synthesized compounds were also screened for their antioxidant activity. Rational studies were carried out by the ABTs method to allow a broader choice of activities. In addition, similar off-compounds were conducted. Molecular docking studies with the CB-Dock server and MD simulations were created with the default settings of the Solution Builder on the CHARMM-GUI server at 150 nm. A good correlation was obtained between the experimental results and the theoretical bioavailability predictions using POM theory.

## Introduction

**Figure 1:**
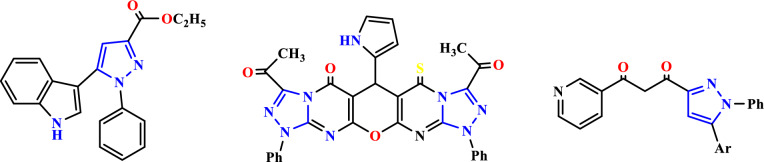
Biologically active compounds have pyrazole rings.

In this context, the pyrazole derivatives skeleton is a fertile source of biologically important molecules possessing a broad spectrum of biological and pharmacological activities such as anti-inflammatory and transformations of condensed heterocyclic derivatives are of theoretical interest for developing new synthetic methods and studying the relationships between chemical structure and reactivity of organic compounds^1^. In addition, pyrazole-based heterocyclic ligands have multiple biological applications. For example, many compounds prepared have high efficiencies as antibacterial or antifungal candidates due to their nitrogen electron and proton acceptor abilities^2^. Furthermore, the presence of the pyrazole nucleus in different structures leads to diversified applications in different areas such as technology, medicine, and agriculture. Furthermore, pyrazole derivatives have arrived an extensive view of researchers through the past few decades due to their highly reactive effects as anti-inflammatory^3^, antiglaucoma^4^, antiviral^5^, antimicrobial^6^, antidiabetic activities^7^. In addition, pyrazole prodrugs have been recorded to maintain significant anticancer activity^8–12^. Pyrazole nucleus is a rare structural stage attractive for combinatorial and medicinal chemistry. In addition, it encloses the most recent reports on a structural variation on pyrazole explaining vital structural activity relationship^13^. Whereas the late-stage explanation of a pyrazole ring through some cycloadditions of previously substituted components is the basis for most substituted pyrazoles^14,15^, direct functionalization has not been examined satisfyingly to date. As analysis on it seems rare, we have been interested in and examined the direct functionalization of pyrazoles through coupling reactions of halogenated analogs taken from commercially available pyrazole^16–19^ and prompted by the observed biological activities of the derivatives mentioned above and in Continuation of our ongoing studies on novel biologically active molecules^20–23^. Nowadays, pyrazole systems, as biomolecules, have attracted more attention due to their interesting pharmacological properties. This heterocycle can be traced in a few well-established drugs belonging to different categories with diverse therapeutic activities. Herein, compound 1 was used as a key intermediate for the synthesis of oxazine, pyrazolotriazinone, and pyrazinopyrimidine derivatives in high yield and purity, to investigate their antimicrobial activity and antioxidant activities (Fig. 1).

With limited facilities to investigate more experimental acceptor abilities, molecular docking became crucial for studying the binding modes and affinities between the prepared compounds and selected biological targets using the lock and key concept^2^. The toxicity predictions and the Lipinski rule of five agreements were determined by POM analysis, one of the well-known approaches to accessing synthetic drugs’ pharmacokinetic properties. It helps identify and indicate the type of pharmacophore site that affects biological activity, wherever there may have been changes in the chemical substitution^24,12^. At the same time, MD studies verified the Molecular docking and toxicity prediction results. The impact of molecular dynamics (MD) simulations in molecular biology and drug discovery has expanded dramatically in recent years. These simulations capture the behavior of proteins and other biomolecules in full atomic detail and at excellent temporal resolution^25^.

## Rational of the work

**Figure 2:**
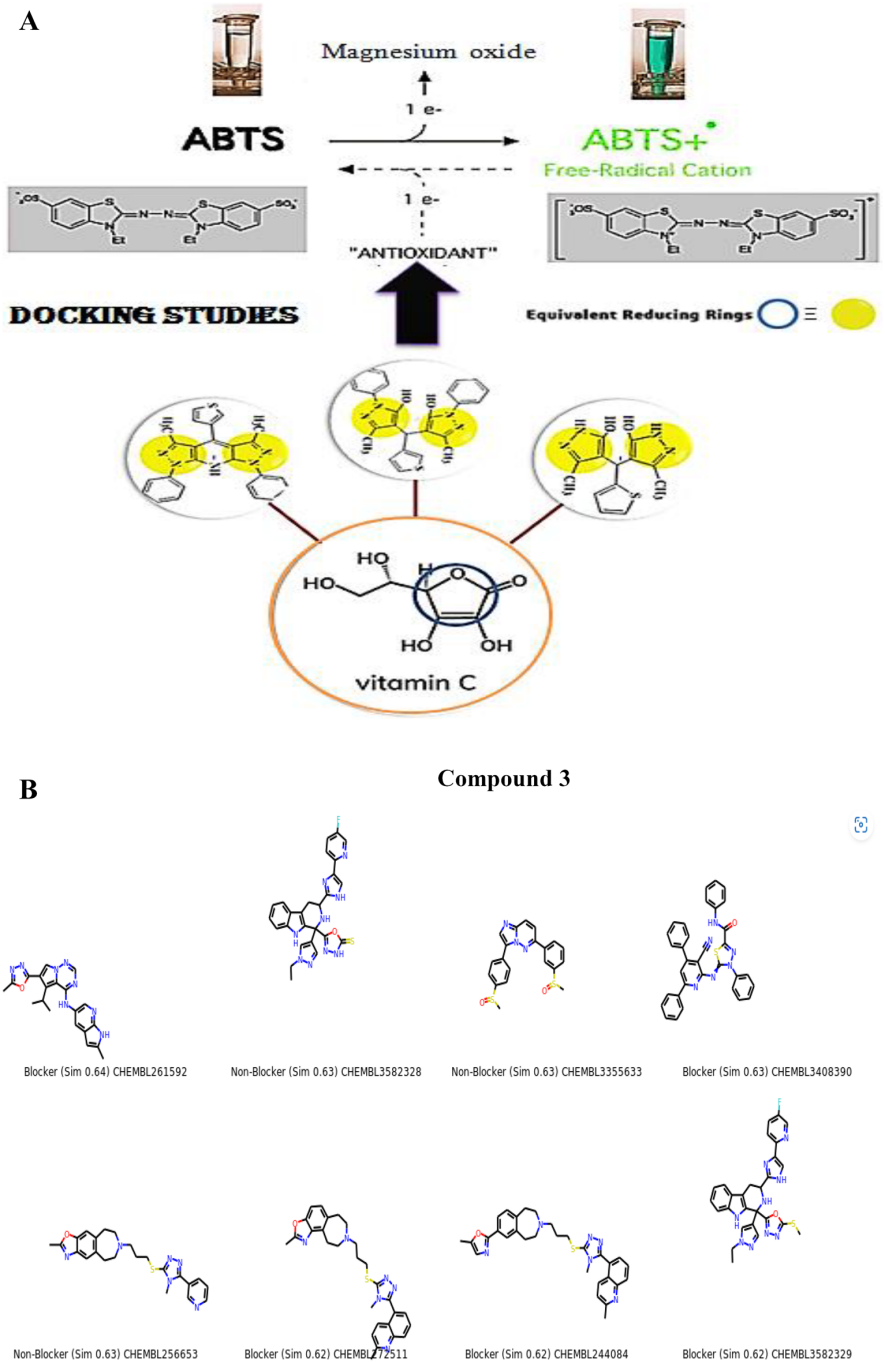

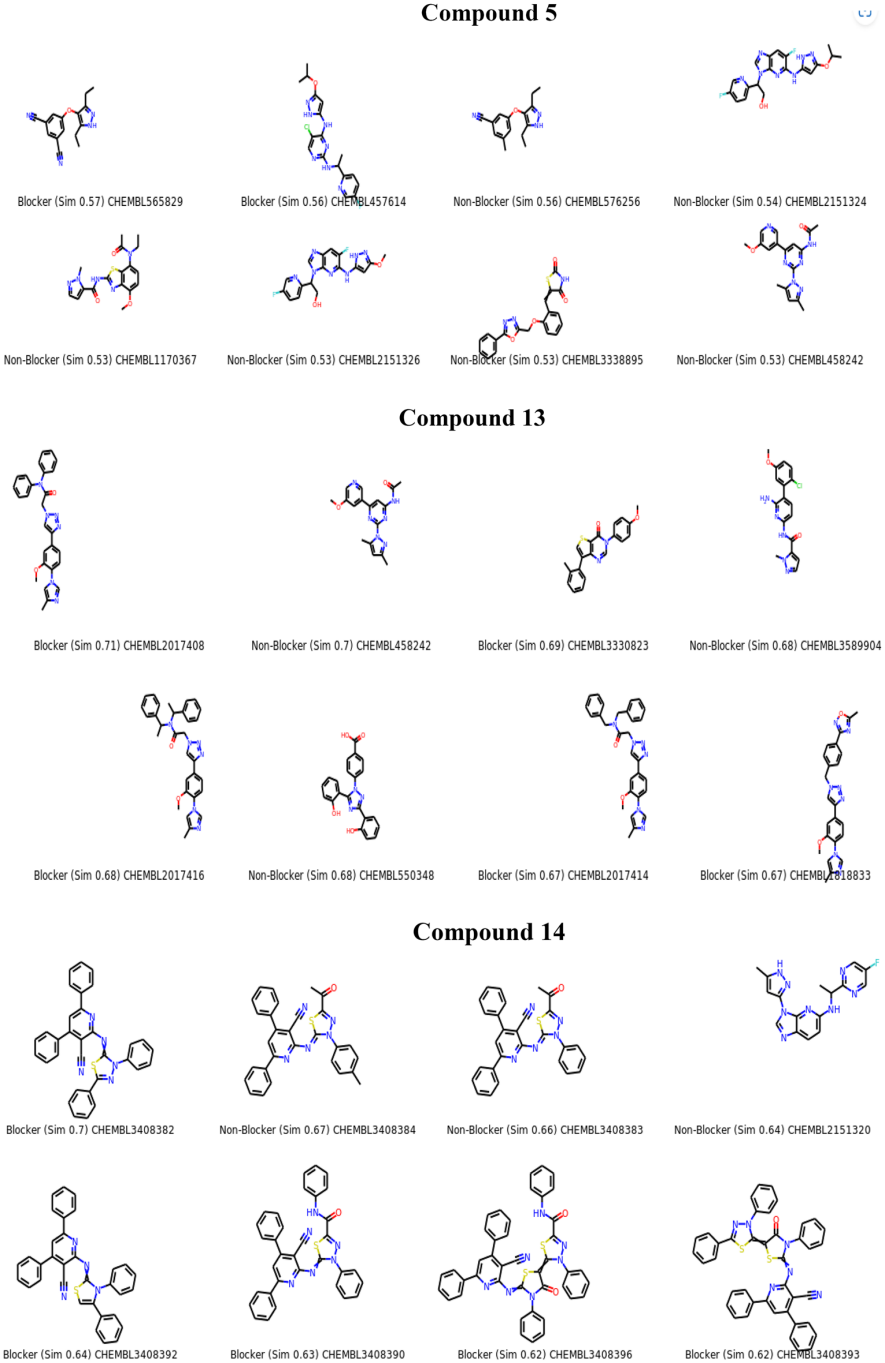

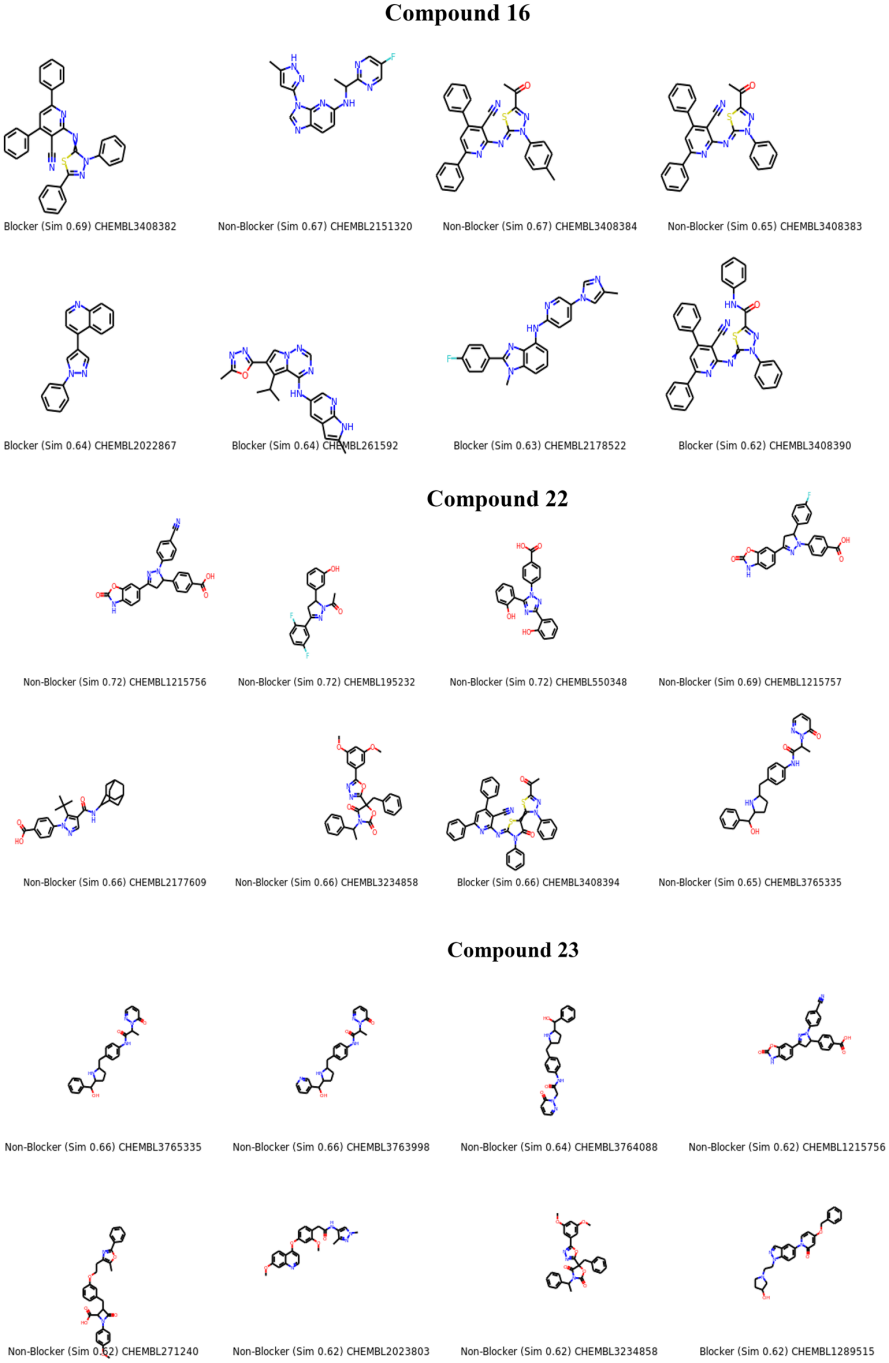
(**A**) Rationale of the synthesized fused pyrazoles derivatives using molecular hybridization. (**B**) The similar off compounds of the new series.

We effectively synthesized a variety of beneficial antioxidants using a molecular hybridization process by choosing to reduce functional groups [Fused Pyrazoles] that are directly related to the reducing ascorbic acid ring Fig. 2A and examined them by the ABTs method. Molecular hybridization was proposed to generate an additive or synergistic impact. In addition, it gives a broader choice of activities^26,27^. Figure 2B represents the similar compounds evaluated and induced by the LabMol server. Chemically similar compounds often bind biologically diverse protein targets, and protein structures do not always recognize identical ligands. Pharmacological and off-target relationships among proteins and a ligand set similarity help to improve the machine learning confidence by interpolating the output prediction equalized by the compound similarity criteria. This pipeline help to improve the predictions of off-target drug effects, reducing the false negative error. Chemical similarity is one of the essential concepts in cheminformatics. One commonly used algorithm to calculate these similarity measures is the 2D Tanimoto algorithm employed here. The resulting Tanimoto coefficient is fingerprint-based, encoding each molecule to a fingerprint “bit” position (MACCS), with each bit recording the presence (“1”) or absence (“0”) of a fragment of the molecule^28,29^.

## Results and discussion

### Chemistry

**Figure 3:**
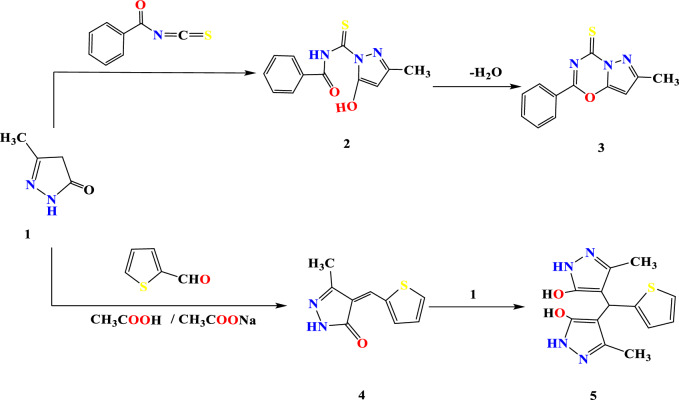
Reaction sequence 1.

Cyclic keto methylene units constitute a precursor for the hetero cyclization system. The present article involved the conversation of a pyrazole-bearing keto methylene system to azolo and amino pyrazole of potential biological activities^30,31^. Pyrazole derivative** 1** added cyclic nucleophilic nitrogen to activated hetero carbon; then intramolecular cyclodehydration formed an oxadiazine ring affording pyrazolo oxadiazine **3.** Figure 3, the structure was potentiated without cyclic carbonyl function frequency and thioxo frequency at 1243 cm^−1^. A multiple for the aromatic system was in the range of 7.34–7.42 ppm, in addition to the pyrazole methylene signal at 2.49 ppm. When thiophene-2-carboxaldehyde was allowed to react with pyrazolone **1** in the presence of acetic acid and sodium acetate, leads to bispyrazole derivative **5**. The formation of bispyrazole is because of the formation of arylidene derivative **4** followed by the conjugate addition of another pyrazolone unit, and the E- and Z-isomers of analogous 5-aryl methylene compounds were identified. It was shown that the olefinic proton of the Z-configured isomers was more deshielded by the 4-oxo group of the thiazole moiety as compared with the E-counterparts and appeared at the lower field (δ ≈ 8.00–8.20 ppm) relative to the E-isomer (δ ≈ 7.50–7.80 ppm) (Fig. 3). The IR spectrum of compound **5** revealed OH, NH peak at 3350, 3550 cm^−1^, and C=N was observed at 1598 cm^−1^. The ^1^HNMR spectra of Compound **5** showed two doublet signals at 7.28 ppm and 7.94 ppm due to thiophenyl –C_4_H and thiophenyl –C_3_H and another at 8.11ppm due to thiophenyl –C_5_H while displayed exchangeable adown field signals at 9.96 and 11.18 ppm for OH and NH protons. Aromatic multiples in the region 7.34–6.92 ppm, in addition to methyl proton signal located at 1.99 ppm, C–O and C=N were shown at 165.45 and 140.78 ppm, respectively.

Contrary to the above result, when thiophene-2-carboxaldehyde was allowed to interact with pyrazolone** 1** in a primary medium resulted in a condensation product **4** affording α, β unsaturated system (Fig. 3). Compound **4** showed a conjugated carbonyl group frequency at 1649 cm^−1^ and a stretching frequency at 1598 cm^−1^ for an exocyclic double bond. The ^1^HNMR spectra showed Olefinic proton at 4.98 ppm in addition to methyl proton at 2.49 ppm and showed two doublet signals at 7.28 ppm and 7.94 ppm due to thiophenyl –C_4_H and thiophenyl –C_3_H and another at 8.11ppm due to thiophenyl –C_5_H while the exchangeable signal at 12.16 ppm for pyrazole NH exchangeable by D_2_O.

**Figure 4:**
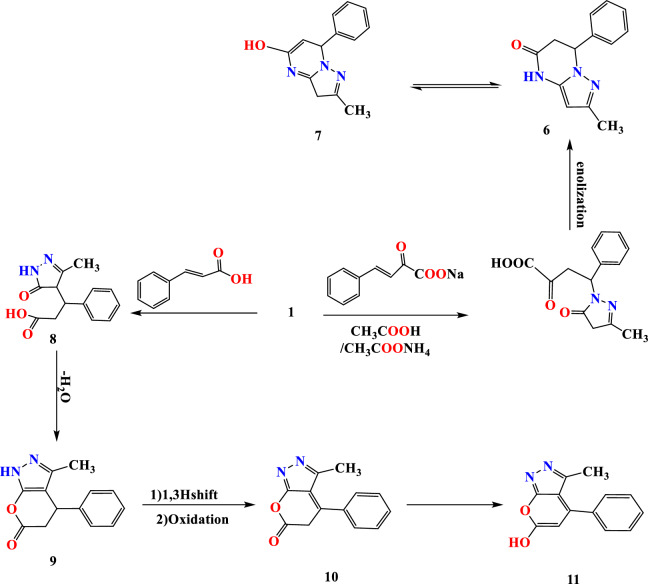
Reaction sequence 2.

The reaction of arylidene sodium pyruvate with pyrazole derivative** 1** in CH_3_COONH_4_ and CH_3_COOH resulted in pyrazolo pyrimidine derivative **7**. The process may proceed via forming aza Michael followed by amination, cyclo dehydration, and subsequent enolization (Fig. 4). ^1^H NMR spectrum of condensed system** 7** revealed exchangeable signal at 13.10 ppm for OH proton in addition to Olefinic proton that located at 6.91 ppm, methylene proton at 2.49 ppm and methyl proton at 3.36 ppm while IR spectrum showed broad band 3350–3550 for (OH) cm^−1^ in addition to a stretching frequency at 1598 cm^−1^ for exocyclic double bond. The cycloaddition of cinnamic acid and pyrazolone 1 was achieved by H_2_SO_4_ as catalyst [activated the carbonyl function in addition to the protonation of pyrazolo nitrogen], resulting in pyran cyclization furnished pyrano pyrazole derivative **11** starting with Michael adduct through **1**, **11** additions followed by elimination of H_2_O. The pyran **11** showed C=N at 1683 cm^−1^, and the ^1^H NMR contained a deshielded signal at 12.35 ppm for OH proton, methyl proton at 2.49, and olefinic proton at 6.53 ppm (Fig. 4).

**Figure 5:**
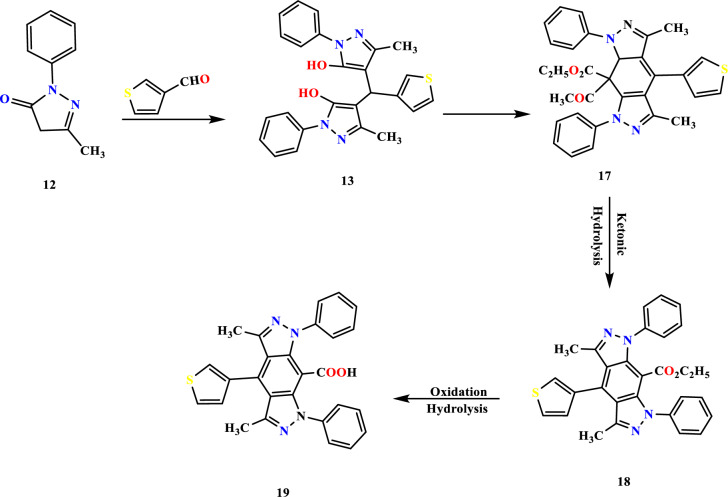
Reaction sequence 3.

*N*-phenyl pyrazolone **12** was reacted with thiophene-3-carboxaldehyde, forming diol derivative **13** (Fig. 5). The OH frequency proved the reaction product 13 at 3460 cm^−1^ and C=N at 1677 cm^−1^. ^1^H NMR revealed a downfield signal at 10.75 ppm for OH and methyl proton at 2.39 ppm. Up on condensation of active methylene of ethyl acetoacetate with pyrazole derivative **13** furnished polycyclic compound **19,** the reaction involves the formation of ketoester **17** ketonic hydrolysis followed by ester hydration and subsequent aromatization (Fig. 5) the chemical structure of the product was potentiated with the presence of carboxylic carbonyl at 1677 cm^−1^ and OH at 3431 cm^−1^, The a presence of exchangeable deshielded signal at 10.78 ppm for carboxylic proton in addition to methyl group was located at 2.41ppm The carbonyl carbon was detected at 164.1

**Figure 6:**
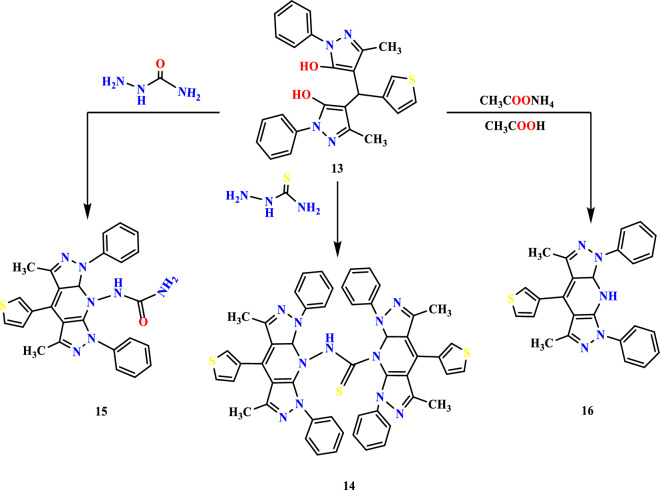
Reaction sequence 4.

Diol **13** was reacted with thiosemicarbazide, producing thioimide product 14 (Fig. 6) via condensing two nitrogen and OH group terminals. Thioimide **14** leads to a stretching frequency at 3426 and 1270 cm^−1^ for NH and C=S, respectively. The thioimide proton signal was downfield at 10.80 ppm, and methyl groups were at 2.43 and 2.49 ppm. When semicarbazide was allowed to be condensed with diol **13**, the urea derivative**15** was formed in Fig. 6. The IR spectrum of compound **15** displayed as bands at 3430, 1679, and 1636 cm^−1^ for NH, C=O, and C=N groups ^1^H NMR revealed NH proton at 10.80 and 9.84 ppm, in addition to pyridine proton that located at 3.35 ppm While in ^13^C The carbonyl carbon signal was showed at 162.78 ppm. Up on heating compound, **13** with ammonium acetate and acetic acid mixture afforded amination followed by pyridine cyclization resulting in di pyrazolo pyridine derivative **16** (Fig. 6) pyridine **16** revealed peaks at 3454 and 1677 cm^−1^ for NH and C=N. Moreover, condensed pyridine **16** showed in ^1^HNMR two doublet signals at 7.28 ppm and 7.94 ppm. This may be due to thiophenyl –C_4_H and thiophenyl –C_3_H and another at 8.11ppm due to thiophenyl –C_5_H and the exchangeable downfield signal at 10.78 ppm for NH and the 3rd proton at 2.46 ppm.

**Figure 7:**
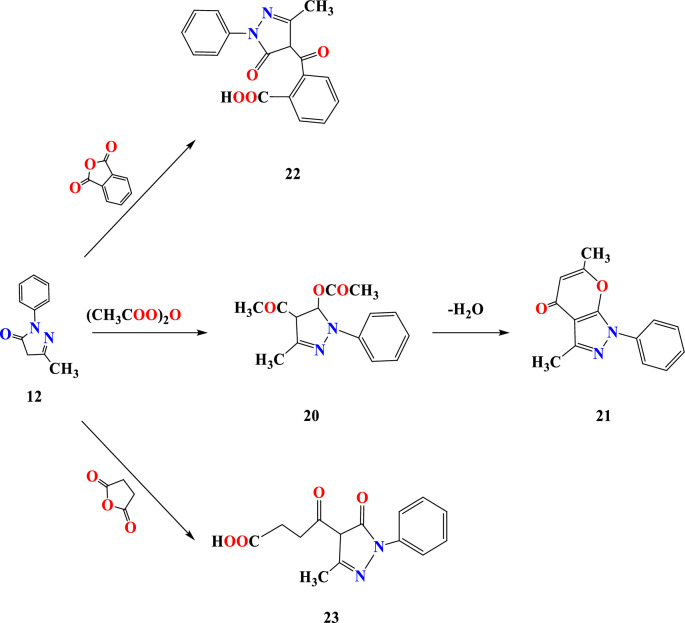
Reaction sequence 5.

Acylation of compound **12** was achieved by reaction with acetic anhydride mixture producing the target **21** displayed carbonyl frequencies at 1677 cm^−1^, ^1^H NMR displayed aromatic multiplet in addition to methyl ester at 2.30 ppm while pyrazole proton was absolved at 2.49 ppm. Pyrone derivative **21** formed ester **20,** followed by acid-catalyzed cyclodehydration (Fig. 7). The phthalic anhydride and N-phenyl pyrazolone **12** with Lewis’s acid resulted in acid derivative **22**. Compound **22** showed 3460, 1756, and 1677 cm^−1^ peaks for OH and carbonyl function, respectively. The exchangeable signal at 10.77 was attributed to the carbonylic proton and the pyrazole proton above and below the plane of the ring that decimated at 3.33 and 2.43 ppm. Succinic anhydrides undergo acylation reaction with compound **12** under an acidic medium to furnish keto acid **23;** the keto acid compound **23** revealed bands at 3457 cm^−1^, 1756 cm^−1^, and 1680 cm^−1^ OH, and carbonyls groups the carbonylic proton signal was located at 10.79 ppm and pyrazole proton was absolved at 2.39 ppm in addition to methylene group at 2.49 and 2.54 ppm.

**Figure 8:**
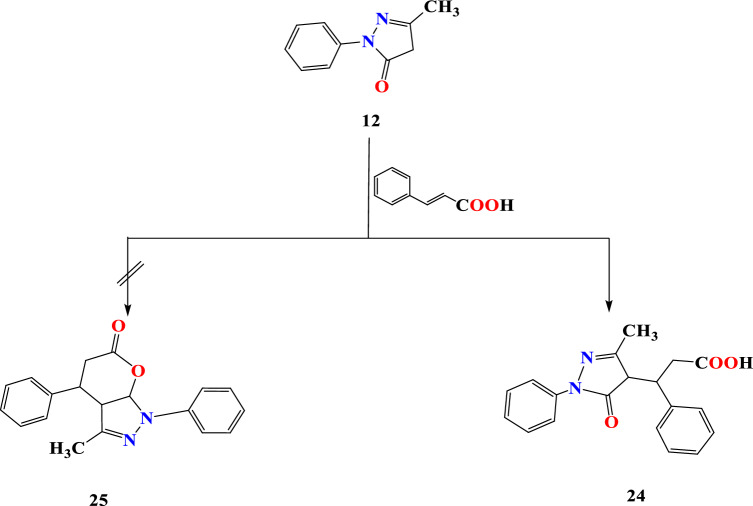
Reaction sequence 6.

Cinnamic acid and N-phenyl pyrazolone **12** undergo conjugate addition, affording acyclic product **24** (Fig. 8), and none of the pyran structure **25** was observed. The IR spectrum **24** leads to OH and carbonyl function at 3461, 1756, 1677, and 1634 ppm, respectively. The carboxyl OH proton was detected at 10.78 ppm in addition to the pyrazolone proton located at 2.43 ppm; the Carbonyl carbon signal was detected at 162.84 ppm.

### Biological activity studies

#### Antioxidant evaluation

**Figure 9:**
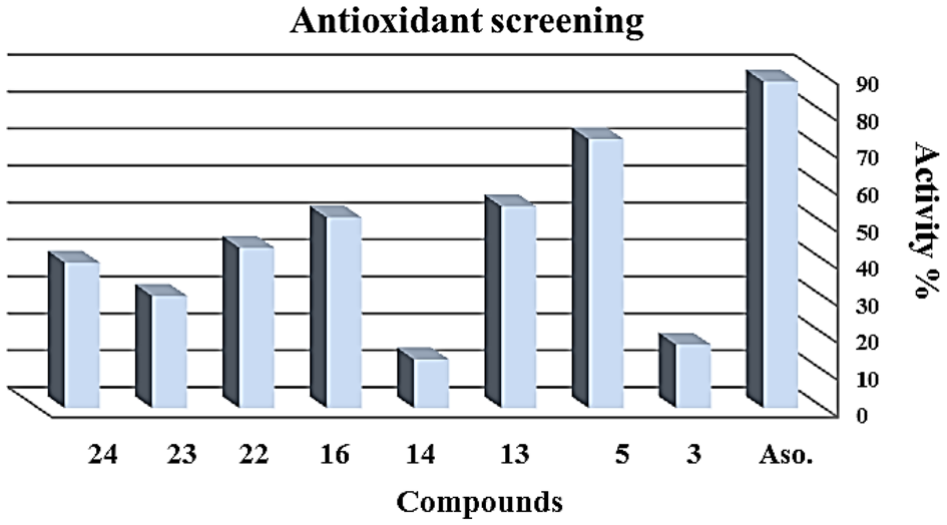
Antioxidant activity screening assay.

The antioxidant activities of the synthesized compounds were determined and listed in Table 1 and Fig. 3. The results revealed that all compounds were found to be potent. Moreover, the results showed that nearly compound **5** was found to have the most potent activity levels. Compounds 13, 14, 16, 22, 23, and 24 also had moderate activity. While compound **14** was found to be the lowest potent level. The following points were noticed. Comparing compound 5 and the other compounds showed that compound **5** indicated that the presence of the 2 OH group was more effective than the other compounds. On the other hand, when C=S in compound **14,** antioxidant activity decreases. While compounds **24**, **22**, **and 23** were more active than compound **14** due to the COOH group’s presence (Fig. 9).

**Table 1:** Antioxidant assay for the tested new compounds.

Method	A.B.T.S Abs(control) − Abs(test)/Abs(control) × 100	
Compounds	Absorbance of samples	% Inhibition
Control of ABTS	0.500	0
Ascorbic acid	0.058	88.4
3	0.414	17.2
5	0.136	72.8
13	0.227	54.6
14	0.434	13.2
16	0.242	51.6
22	0.283	43.4
23	0.348	30.4
24	0.303	39.4

#### Antimicrobial studies

**Figure 10:**
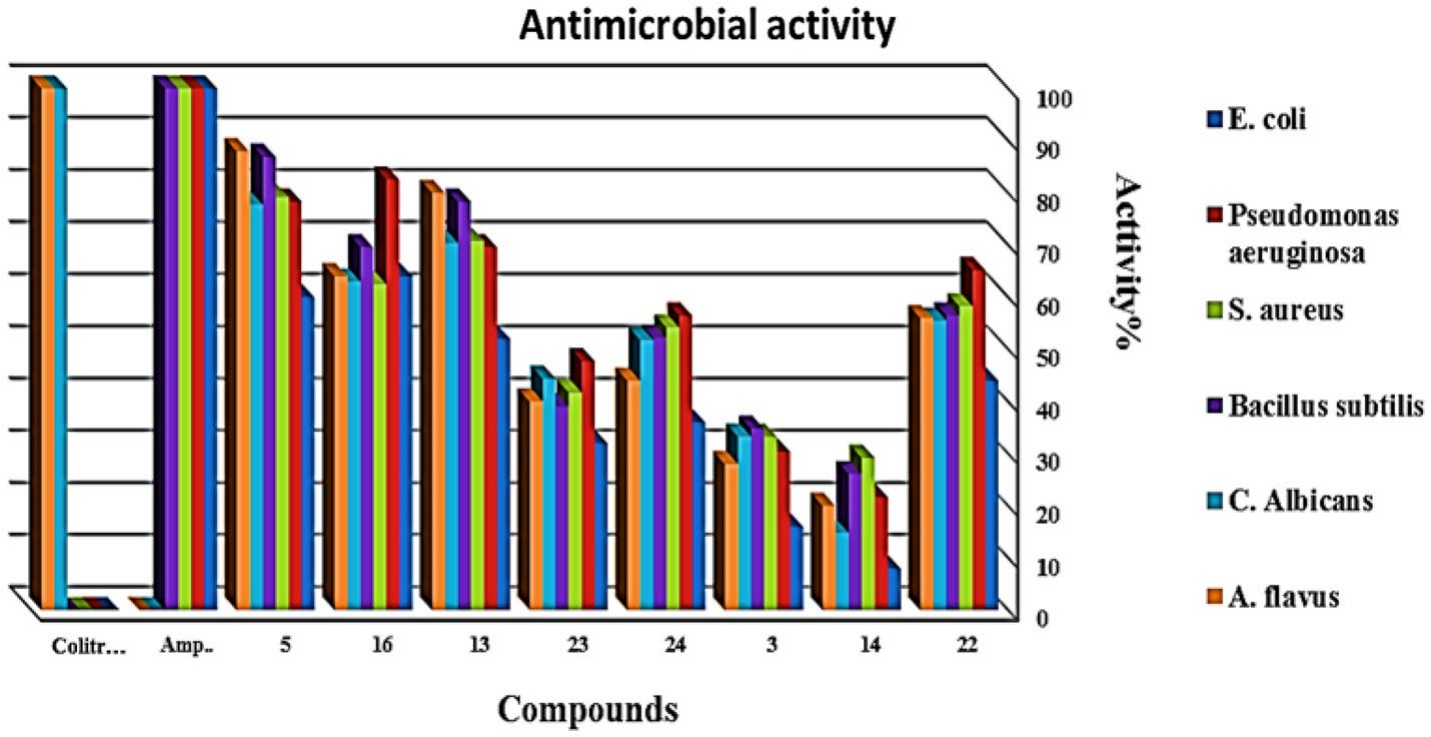
In‐vitro antibacterial and antifungal activity screening assay.

All the tested compounds have antibacterial activity against two-gram positive bacteria (*Staphylococcus aureus*, *Bacillus subtilis*) and Gram-negative bacteria (*Escherichia coli*, *Pseudomonas aeruginosa*). In addition, the antifungal activities of the compounds were tested against two fungi (Candida albicans, Aspergillus flavus) from (Table 2) the most reactive is compound 4,4′-(thiophen-2-ylmethylene)bis(3-methyl-1H-pyrazole-5-ol) (**5**) and the lowest is compound N-(3,5-dimethyl-4-(thiophen-3-yl)dipyrazolo[3,4-b:4′,3′-e]pyridin-8(3H)-yl)-3,5-dimethyl-4-(thiophen-3-yl)-5,7a-dihydrodipyrazolo[3,4-b:4′,3′-e]pyridine-8(3H)-carbothioamide (**14**) (Fig. 10).

**Table 2:** In‐vitro antibacterial and antifungal screening of the newly synthesized compounds.

Compound	*E. coli*		*Pseudomonas aeruginosa*		*S. aureus*		*Bacillus subtilis*		*C. albicans*		*A. flavus*	
	Diameter of inhibition zone (mm)/activity index %											
3	4	16	7	30.4	8	33.3	8	34.8	9	33.3	7	28
5	15	60	18	78.3	19	79.2	20	86.9	21	77.8	22	88
13	13	52	16	69.6	17	70.8	18	78.3	19	70.4	20	80
14	2	8	5	21.7	7	29.2	6	26.1	4	14.8	5	20
16	16	64	19	82.6	15	62.5	16	69.6	17	63	16	64
22	11	44	15	65.2	14	58.3	13	56.5	15	55.5	14	56
23	8	32	11	47.8	10	41.7	9	39.1	12	44.4	10	40
24	9	36	13	56.5	13	54.2	12	52.2	14	51.8	11	44
Ampicillin	25	100	23	100	24	100	23	100	N.A.	–	N.A.	–
Colitrimazole-ole	N.A.	–	N.A.	–	NA.	–	N.A.	–	27	100	25	100

**Figure 11:**
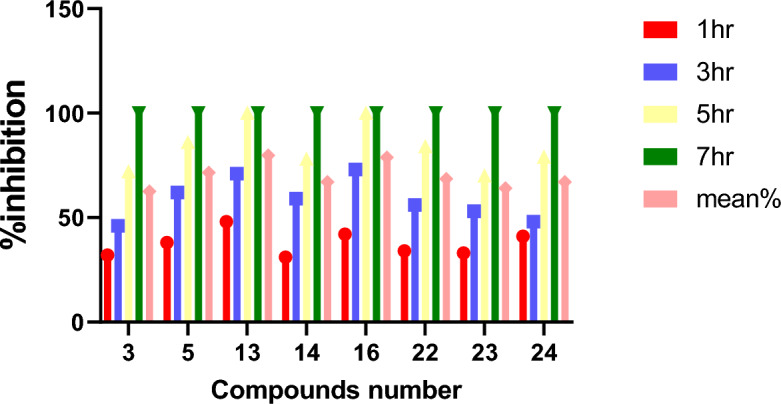
The mean percent of bactericidal in time by *hr.

**Figure 12:**
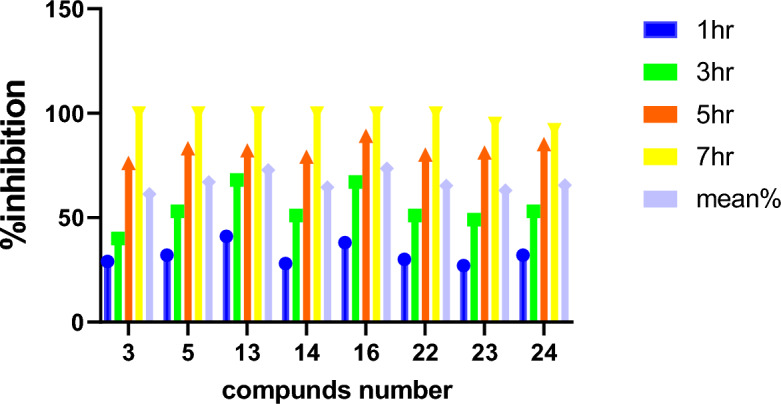
The mean percent % of bactericidal in time by *hr.

**Figure 13:**
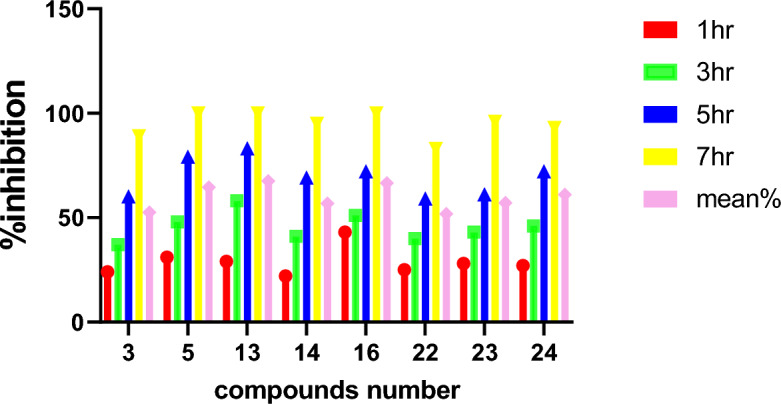
The mean percent % of bactericidal in time by *hr for compound 13.

Another method was used to determine the Inhibition by recognition time to achieve maximum Inhibition, which means antimicrobial studies were carried out by measuring the growth inhibition time and taking the mean of Inhibition by time method^32,33^. This method indicates the mean time of Inhibition. This test was carried out on the highly active compound observed in both the MIC test and the computational studies. Table 3 and Fig. 11 showed in-vitro experiments for the mean antimicrobial effects on *G-positive* bacteria *S. aureus*, revealing. The mean antimicrobial effects were calculated. The same procedure was repeated on *G-Negative bacteria E. coli*, and the results are indexed in Table 4 and Fig. 12. At the same time, in-vitro experiments for the mean of antifungal effects on *C. Albicans* and the results are indexed in Table 5 and Fig. 13. Bacteria have developed resistance strains against currently available antimicrobial agents and synthesize novel antimicrobials with less toxicity and more potent effects in less time. The minimal inhibitory concentration for some of the newly synthesized compounds showed highly significant activity**.** Among the screened compounds, 5, 13, and 16 exhibited intense antimicrobial activity^34^.

**Table 3:** The mean percent of bactericidal in time by *h.

Compound/time	1 h	3 h	5 h	7 h	The mean (%)
3	32	46	72	100	62.5
5	38	62	86	100	71.5
13	48	71	100	100	79.8
14	31	59	78	100	67.0
16	42	73	100	100	78.8
22	34	56	84	100	68.5
23	33	53	70	100	64
24	41	48	79	100	67

**Table 4:** The mean percent % of bactericidal in time by *h.

Compound/time	1 h	3 h	5 h	7 h	The mean (%)
3	29	40	76	100	61.3
5	32	53	83	100	67.0
13	41	68	82	100	72.8
14	28	51	79	100	64.5
16	38	67	89	100	73.5
22	30	51	80	100	65.3
23	27	49	81	95	63
24	32	53	85	92	65

**Table 5:** The mean percent of bactericidal in time by *h.

Compound/time	1 h	3 h	5 h	7 h	The mean (%)
3	24	37	60	89	52.5
5	31	48	79	100	64.5
13	29	58	83	100	67.5
14	22	41	69	95	56.8
16	43	51	72	100	66.5
22	25	40	59	83	51.8
23	28	43	61	96	57
24	27	46	72	93	61

### Minimal inhibitory concentration (MIC)

In general, our synthesized derivatives showed activity against the tested Gram-positive bacteria and the Gram-negative bacteria. The data obtained from the previous two methods showed that compounds 13 and 16 were the most active. Compounds **13** and **16** were investigated again using the MIC test against two-gram positive bacteria (*Staphylococcus aureus*, *Bacillus subtilis*) and Gram-negative bacteria (*Escherichia coli*, *Pseudomonas aeruginosa*) (Table 6).

**Table 6:** Antimicrobial activity of compounds.

Compound	*E. coli*	*Pseudomonas aeuginosa*	*Bacillus subtilis*	*S. aureus*
	% Inhibition			
13	36	19	31	23
16	43	24	39	34
Control: DMSO	0	0	0	0
Amikacin	39	30	38	28
Penicillin	0	20	0	18

### Computational studies

#### Molecular docking

**Figure 14:**
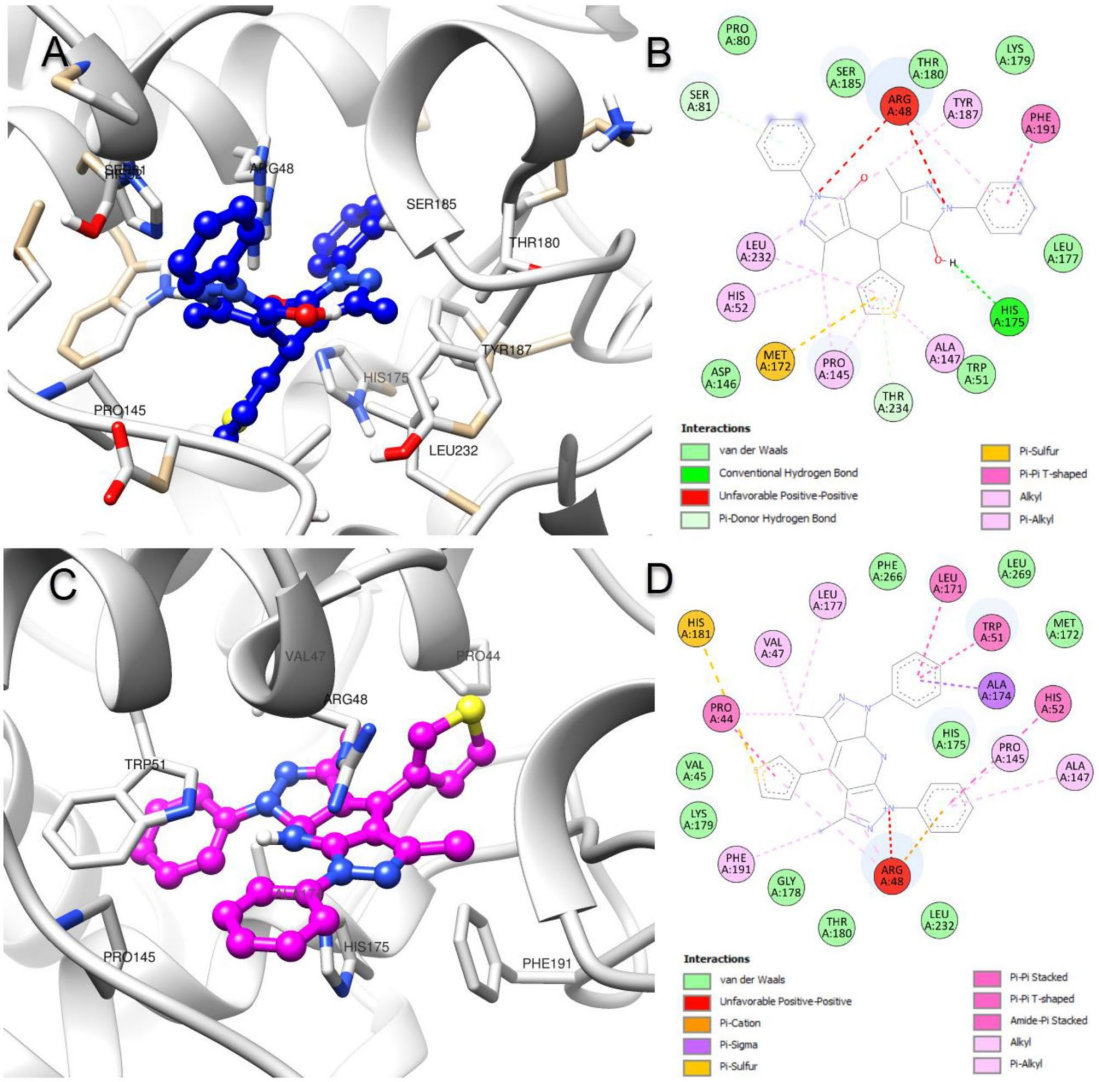
(**A**,**B**) Molecular docking binding poses and protein–ligand schematic interaction diagram of compounds **13** and target protein cytochrome c peroxidase (CCP), and (**C**,**D**) compounds **16** and CCP complex (PDB ID: 2X08).

Molecular docking studies were used to predict in silico how newly synthesized compounds interact with target proteins. In this context, active compounds 13 and 16 interactions with the cytochrome c peroxidase (CCP) enzyme selected as the target protein were analyzed. Compounds **13** and **16**, given in Table 7, produced − 9.1 kcal/mol and − 9.6 kcal/mol interaction energies, while CCP’s cocrystal ligand ascorbic acid gave − 5.9 kcal/mol interaction energies. As shown in Table 7, atomic-level interactions, interaction distances, and types of CCP & **13** and CCP & **16** complexes were analyzed. Both compounds exhibited more potent interactions with the target protein CCP than the standard ascorbic acid. As given in Fig. 14, compound **13** conferred an H bond with His75, while compounds **13** and **16** formed an unfavorable positive-positive interaction with Arg48.

**Table 7:** Protein–ligand interaction energies and details of the compounds **13** and **16** with target protein cytochrome c peroxidase (CCP).

Complex	Name	Distance (Å)	Category	Types
CCP & 13 (− 9.1 kcal/mol)	H-His175:NE2	2.55	Hydrogen bond	Conventional hydrogen bond
	Ser81:OG	3.92		Pi–donor hydrogen bond
	Thr234:OG1-LIG	4.06		Pi–donor hydrogen bond
	Met172:SD-LIG	5.41	Hydrophobic	Pi–sulfur
	Phe191-LIG	5.18		Pi–Pi T-shaped
	LIG:C-Pro145	4.22		Alkyl
	LIG:C-Leu232	3.97		Alkyl
	His52-LIG:C	4.47		Pi–alkyl
	Tyr187-LIG:C	4.78		Pi–alkyl
	LIG-Arg48	4.32		Pi–alkyl
	LIG-Pro145	4.65		Pi–alkyl
	LIG-Ala147	4.62		Pi–alkyl
	LIG-Leu232	5.27		Pi–alkyl
CCP & 16 (− 9.6 kcal/mol)	Arg48:NH2-LIG	3.70	Hydrogen bond; electrostatic	Pi–cation; Pi–donor hydrogen bond
	Ala174:CB-LIG	3.76	Hydrophobic	Pi–sigma
	LIG:S-His181	5.81		Pi–sulfur
	Trp51-LIG	3.76		Pi–Pi stacked
	Trp51-LIG	4.75		Pi–Pi stacked
	His52-LIG	5.89		Pi–Pi T-shaped
	Pro44:C,O;Val45:N-LIG	4.36		Amide–Pi stacked
	Leu171:C,O;Met172:N-LIGS	4.48		Amide–Pi stacked
	LIG:C-Pro44	5.04		Alkyl
	LIG:C-Val47	4.02		Alkyl
	LIG:C-Arg48	4.16		Alkyl
	LIG:C-Leu177	4.88		Alkyl
	Phe191-LIG:C	4.59		Pi–alkyl
	LIG-Pro145	5.00		Pi–alkyl
	LIG-Ala147	5.46		Pi–alkyl
	LIG-Pro44	5.19		Pi–alkyl
	LIG-Arg48	4.61		Pi–alkyl
	LIG-Leu171	5.42		Pi–alkyl

#### Molecular dynamics simulations

**Figure 15:**
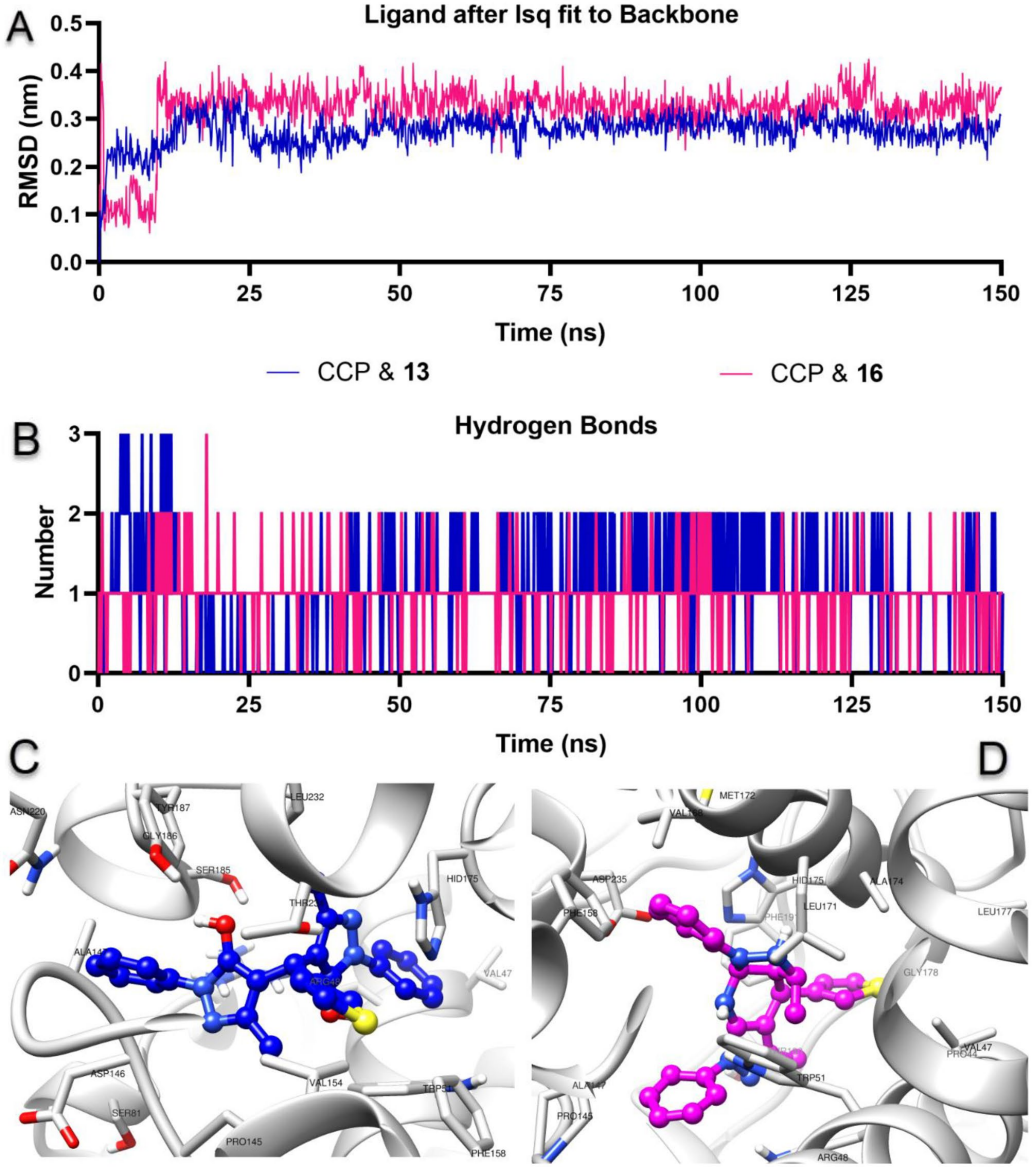
Molecular dynamics simulations of compounds **13** and **16** with cytochrome c peroxidase (CCP). (**A**) The root means square deviation (RMSD) plot shows the ligand’s conformational changes at the protein active site. (**B**) The number of H bonds formed between the CCP and compounds **13** and **16** throughout 150 ns. (**C**,**D**) Binding poses of CCP & **13** and CCP & **16** complexes at the end of 150 ns, respectively.

Molecular dynamics (MD) simulations were performed to investigate the stability of CCP & **13** and CCP & **16** complexes obtained with AutoDock Vina. Root means square deviation (RMSD) analysis and H bond analysis between protein and ligand, two important trajectory analysis parameters of MD simulation, carried out for 150 ns duration, were performed. Fitting ligands performed RMSD analysis to protein backbone atoms to analyze the changes of compounds at the target protein active site. As shown in Fig. 15A, the CCP & **13** and CCP & **16** complexes remained stable in the active pocket after the first 20 ns of pre-MD simulation with mean values below 0.4 nm and 0.27 ± 0.03 and 0.32 ± 0.05 nm in both compounds, respectively. In addition, H bond number measurement was also measured over time, another way of analyzing protein–ligand interactions. After the first 20 ns of pre-MD simulation, as shown in Fig. 15B, there was a continuous H bond formation ranging from one to two between compounds **13** and **16** with CCP Binding mode analysis was performed, as shown in Fig. 15C,D, to analyze protein–ligand interactions at the end of 150 ns and compare them with the docking pose. When the docking pose and the binding poses at the end of the MD simulation were compared, it was concluded that both compounds formed strong and stable interactions at the CCP active site.

**Figure 16:**
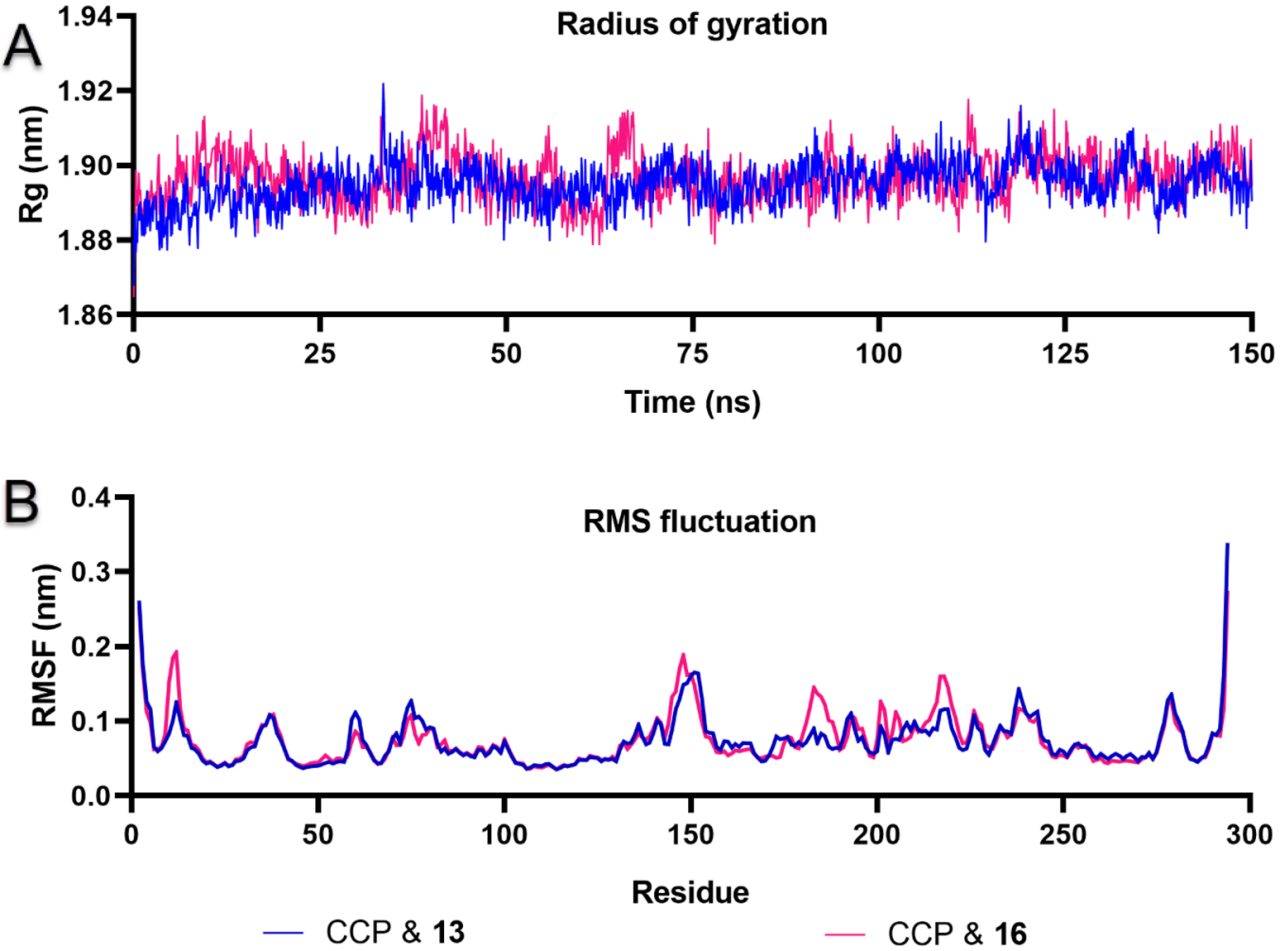
(**A**) Radius of gyration (Rg) plot describing the compactness of the protein–ligand complexes CCP & **13** and CCP & **16**, and (**B**) root mean square fluctuation (RMSF) plot showing the flexibility and mobility of the protein per residue for 150 ns.

In the second MD trajectory analysis section, the radius of gyration (Rg) analysis was performed to explain the compactness of protein–ligand complexes. As shown in Fig. 16A, both CCP & **13** and CCP & **16** protein–ligand complexes were extremely stable around 1.90 nm for 150 ns. Root mean square fluctuation (RMSF) measurements were made to examine the flexibility of the protein structure. As shown in Fig. 16B, the RMSF value was below 0.2 nm except for the protein C and N terminal residues. Active site residues around His175, Leu171, Met172, and Leu177 fluctuated below 0.1 nm for both protein–ligand complexes. MD animation videos in Supporting Information Videos S1 and S2 were created at 150 ns.

## POM study

The POM theory is among the most effective platforms because it can fundamentally and efficiently process all organic and organometallic compounds. Tanks of this theory, the design, optimization, and identification of pharmacophore sites (anticancer/antibacterial/antifungal/antiparasitic/ antiviral) depend on every site’s physical and chemical properties and Mulliken charges analysis of heteroatoms^35^.

The cLogP and TPSA (Molecular Polar Surface Area) were calculated by using the Osiris program (Tables 7, 8). These two parameters have been considered among the essential descriptors to determine drug absorption, involving each drug molecule's bioavailability, intestinal absorption, and lipophilicity^36^.

**Table 8:**
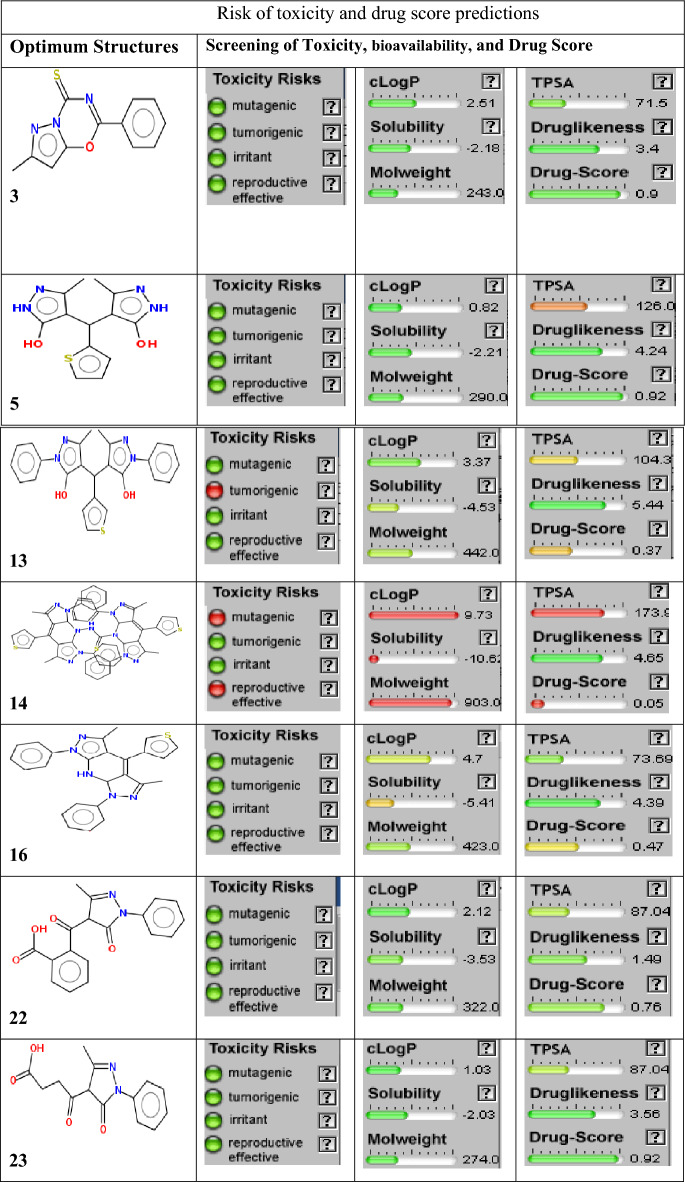
Risk of toxicity and drug score predictions.

**Figure 17:**
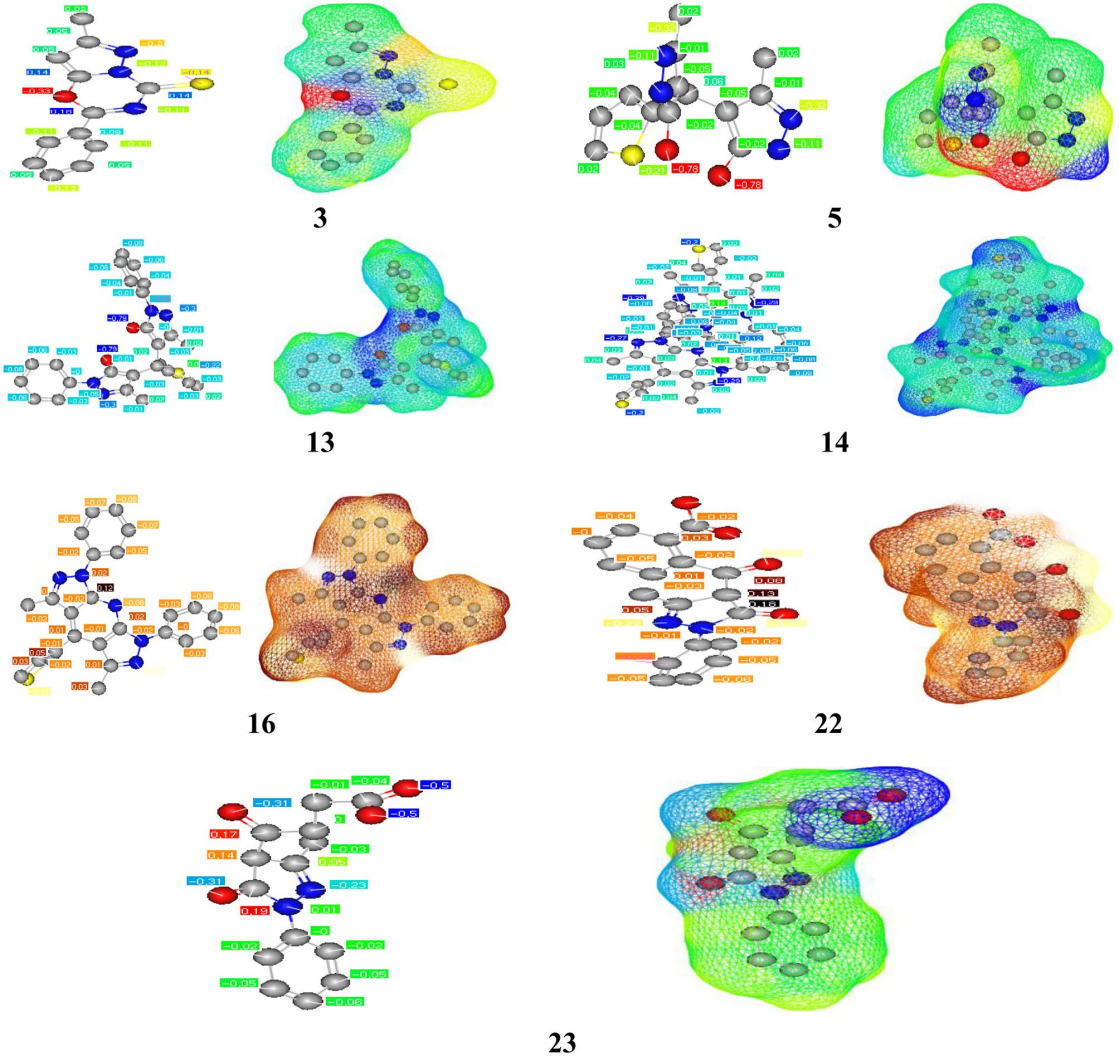
Atomic charges of tested compounds.

**Figure 18:**
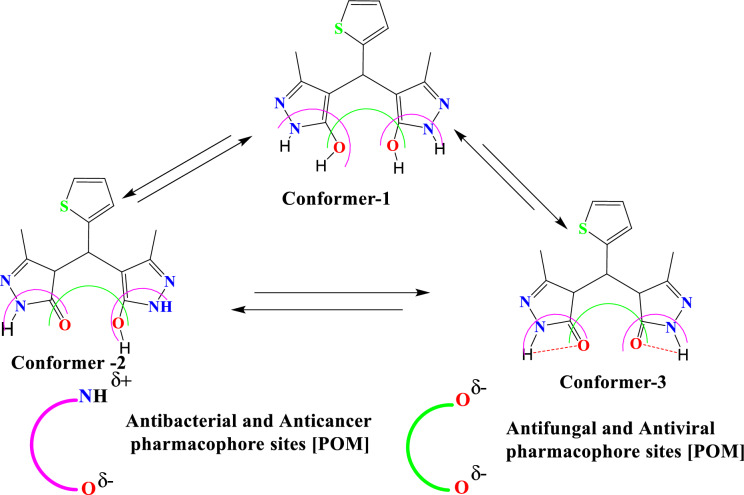
Identification of antibacterial and antifungal pharmacophore sites.

The cLogP and TPSA calculations were done for all prepared compounds by POM programs. The predicted cLogP values are less than 5 for all tested molecules except compound 14 has exhibited a clogP of more than 9. Therefore, the values for all studied compounds are less than 5, except for 14. That represents the higher limit for the drug, which can pass into bio-membranes and react with all pockets conforming to the five rule (Lipinski’s rule). Based on cLogP values, the prepared compounds are expected to give adequate water solubility and bioavailability (Tables 7, 8). In addition, most of the tested compounds do not exhibit any hazards or toxicities. Compounds 13 and 14 have shown tumorigenic, mutagenic, and reproductive effects of risks of toxicities. However, all tested compounds have generally illustrated great safety and excellent agreement with experimental results and are in an acceptable range compared to the five rules (Tables 8, 9). (5, 16, 13, 22 Antibacterial) We have remarked on the presence of many pharmacophore sites (Fig. 17). Most are (–NH^+^; –O^δ−^) antibacterial sites. Next are (–O^δ−^; –O^δ−^) antifungal and pharmacophore sites (Fig. 18)^27,37^.

**Table 9:** Physicochemical properties and drug likeness prediction of all prepared compounds.

Compd.	Physicochemical properties				Drug likeness					
	*Mw*	*O/NH*	*NV*	*VOL*	*GPCRL*	*ICM*	*KI*	*NRL*	*PI*	*EI*
3	243	0	0	202	− 0.75	− 0.60	− 0.77	− 1.06	− 0.79	− 054
5	290	4	0	245	− 0.68	− 0.72	− 0.61	− 0.88	− 0.99	− 0.45
13	442	2	0	388	− 0.23	− 0.16	− 0.38	− 0.38	0.36	− 0.26
14	904	1	3	777	− 3.36	− 3.68	− 3.66	− 3.68	− 3.02	− 3.54
16	423	1	0	374	− 0.09	− 0.34	− 0.51	− 0.45	− 0.39	− 0.18
22	322	1	0	279	− 0.42	− 0.42	− 0.93	− 0.43	− 0.50	− 0.34
23	274	1	0	241	− 0.51	− 0.46	− 1.35	− 0.41	− 0.57	− 0.29

*TPSA* total polar surface area, *O/NH* O–HN interaction, *VIOL* number of violation, *VOL* volume, *GPC* GPCR ligand, *ICM* ion channel modulator, *KI* kinase inhibitor, *NRL* nuclear receptor ligand, *PI* protease inhibitor, *EI* enzyme inhibitor.

## Materials and methods

All chemicals were purchased from Sigma-Aldrich (Taufkirchen, Germany), and all solvents were purchased from El-Nasr Pharmaceutical Chemicals Company (analytical reagent grade, Egypt). All chemicals were used as supplied without further purification. The melting points were measured by a digital Electrothermal IA 9100 Series apparatus Cole-Parmer, Beacon Road, Stone, Staffordshire, ST15 OSA, UK) and were uncorrected. The spectral analysis was performed at KAUST and Mansura Univesity laboratories C, H, and N analyses on a PerkinElmer CHN 2400. In addition, ^1^H and ^13^C NMR spectra were recorded at KAUST on a Bruker 800 MHz NMR Spectrometer using tetramethylsilane (TMS) as the internal standard, chemical shifts were expressed in *δ* (ppm), and DMSO-*d*_6_ was used as the solvent.

### 7-Methyl-2-phenyl-4H-pyrazolo[5,1-b] [1,3,5] oxadiazine-4-thione (3)

A mixture of pyrazole **1** (0. 01 mol) and benzoyl isothiocyanate (0.02 mol) in dioxane (20 ml) and triethylamine (5 drops) was heated under reflux for 6 h. The reaction mixture was cooled, and the separated solid product was filtered off, dried, crystallized from ethanol, and given compound** 3** as orange crystals in 65% yield, m.p.30 °C. IR (KBr, ν, cm^−1^): 3032 (aromatic CH), 2995 (aliphatic CH), 1616 and 1594 (2C=N stretch), 1243 (C=S stretch), 1109 (C=S stretch). ^1^H NMR(DMSO-d_6_, 400 MHz): δ = 2.49 (s, 3H, CH_3_), 4.34 (s, H, =CH), 7.34–7.42 (m, 5H, ArH’s) ppm. ^13^C NMR (DMSO-d_6_) δ = 136.11, 129.03, 128.75, 128.28, 112.96, 39.50, 37.02 ppm. Anal. Calcd for C_12_H_9_N_3_OS (243.28): C, 59.20; H, 3.69; N, 17.25; S, 13.20. Found: C, 59.24; H, 3.73; N, 17.27; S, 13.18%.

### (Z)-5-Methyl-4-(thiophen-2-ylmethylene)-2,4-dihydro-3H-pyrazol-3-one (4)

A mixture of pyrazole **1** (0. 01 mol) and thiophene-2-carboxaldehyde (0.01 mol) in piperidine (10 ml) was fused for an hour. The reaction mixture was cooled, and the separated solid product was filtered off, crystallized from ethanol, and compound **4** as brown crystals in 70% yield, m.p.230 °C. IR (KBr, ν, cm^−1^): broadband 3350–3550(NH stretch), 3103(aromatic CH), 2928 (aliphatic CH), 1649 (C=O amide), 1598 (C=N), 1071 (C–S stretch). ^1^H NMR (DMSO-d_6_, 400 MHz) δ = 2.49 (s, 3H, CH_3_), 4.98 (s, H, olefinic proton), 7.28 (d, 1H, *J* = 3.6 Hz, thienyl-C_3_/*H*), 7.94 (dd, 1H, thienyl-C_4_/*H*), 8.11 (d, d, 1H, *J* = 5.2 Hz, thienyl-C_5_/*H*), 6.91–7.37 (m, 5H, ArH’s), 12.16 (s, H, NH exchangeable by D_2_O) ppm. ^13^C NMR (DMSO-d_6_) δ = 166.8, 145.90, 139.31, 131.81, 127.80, 127.51, 127.31, 126.01, 15.01 ppm. Anal. Calcd for C_9_H_8_N_2_OS (192.24): C, 56.29; H, 4.15; N, 14.62; S, 16.75. Found: C, 56.23; H, 4.19; N, 14.57; S, 16.68%.

### 4,4′-(Thiophen-2-ylmethylene) bis(3-methyl-1H-pyrazol-5-ol) (5)

A mixture of pyrazole **1** (0. 02 mol) and thiophene-2-carboxaldehyde (0.01 mol) in acetic acid (30 ml) and sodium acetate (0.01 mol) was heated under reflux for 6 h. The reaction mixture was cooled, and the separated solid product was filtered off, dried, crystallized from ethanol, and given compound **5** as black crystals in 76% yield, m.p. above 300 °C. IR (KBr, ν, cm^−1^): broadband 3350–3550 (2NH and 2OH stretch), 2922 (aliphatic CH), 1598 (C=N stretch), 1296 (S–C stretch). ^1^H NMR(DMSO-d_6_, 400 MHz) δ = 1.99 (s, 6H, 2CH_3_), 4.96 (s, 1H, =CH), 7.28 (d, 1H, *J* = 3.6 Hz, thienyl-C_3_/*H*), 7.94 (dd, 1H, thienyl-C_4_/*H*), 8.11 (d, d, 1H, *J* = 5.2 Hz, thienyl-C_5_/*H*), 9.96 and 10.34 (s, 2H, OH), 11.18 (s, 2H, NH exchangeable by D_2_O) ppm. ^13^C NMR (DMSO-d_6_) δ = 184.36, 165.45(C–O), 161.67, 159.02, 149.37, 140.87(C=N), 138.15, 136.63, 135.92, 128.31, 126.74, 125.25, 124.69, 121.44, 99.69, 46.31, 39.50, 29.01, 21.09, 13.02, 12.85, 11.36, 10.29 ppm. Anal. Calcd for C_13_H_14_N_4_O_2_S (290.34); C, 53.85; H, 4.91; N, 19.36; S, 11.12. Found: C, 53.78; H, 4.86; N, 19.30; S, 11.04%.

### 2-Methyl-7-phenyl-3,7-dihydropyrazolo[1,5-a] pyrimidin-5-ol (7)

A mixture of pyrazole **1** (0. 01 mol) and benzylidene sodium pyruvate (0.02 mol) in acetic acid (30 ml) and ammonium acetate (0.01 mol) was heated under reflux for 6 h. The reaction mixture was cooled, and the separated solid product was filtered off, dried, crystallized from ethanol, and given compound **7** as orange crystals in 55% yield, m.p.240 °C. IR (KBr, ν, cm^−1^): broadband 3350–3550 (OH stretch), 2916 (aliphatic CH), 1598 (C=N). ^1^H NMR (DMSO-d_6_, 400 MHz) δ = 2.22 (s, 3H, CH_3_), 2.49 (s, 2H, methylene), 3.36 (s, 1H, CH), 6.91 (s, H, olefinic proton), 6.91–7.82 (m, 5H, ArH’s), 8.43 (d, H, ethylene), 13.10 (s, H, OH) ppm. ^13^C NMR (DMSO-d_6_) δ = 156.10, 154.30, 150.90, 138.90, 128.41, 127.80, 127.00, 123.80, 56.60, 27.00, 25.90, 23.10 ppm. Anal. Calcd for C_13_H_13_N_3_O (227.27): C, 68.65; H, 5.73; N, 18.42; Found: C, 68.70; H, 5.77; N, 18.49%

### 3-Methyl-4-phenylpyrano[2,3-c] pyrazol-6-ol (11)

A mixture of pyrazole **1** (0. 01 mol) and cinnamic acid (0.01 mol) in dioxane (20 ml) and conc sulfuric acid (5 drops) was heated under reflux for 6 h. The reaction mixture was cooled, and the separated solid product was filtered off, dried, crystallized from ethanol, and given compound **11** as buff crystals in 70% yield, m.p.118 °C. IR (KBr, ν, cm^−1^): broadband 3450–3500 (OH str), 3063 (CH aromatic), 2971 (CH aliphatic), 1683 (C=N stretch). ^1^H NMR (DMSO-d_6_, 400 MHz) δ = 2.49 (s, 3H, CH_3_), 6.53 (s, H, =CH), 7.39–7.67 (m, 5H, ArH’s), 12.35 (s, H, OH) ppm. ^13^C NMR (DMSO-d_6_) δ = 164.40, 161.71, 152.11, 151.70, 128.40, 127.80, 127.50, 127.30, 126.80, 83.70, 14.50 ppm. Anal. Calcd for C_13_H_10_N_2_O_2_ (226.24): C, 69.09; H, 4.51; N, 12.48. Found: C, 69.02; H, 4.46; N, 12.38%.

### 4,4′-(Thiophen-3-ylmethylene) bis(3-methyl-1-phenyl-1H-pyrazol-5-ol) (13)

A mixture of pyrazole **12** (0. 02 mol) and thiophene-3-carboxaldehyde (0.01 mol) in sodium metal (0.02 g) dissolved in ethanol (20 ml) was heated under reflux for 4 h. The reaction mixture was cooled, and the separated solid product was filtered off, dried, crystallized from ethanol, and compound **13** as brown crystals in 59% yield, m.p.102 °C. IR (KBr, ν, cm^−1^): broadband 3440–3520 (2OH stretch), 2920 (aliphatic CH), 3069 (aromatic CH), 1677(C=N stretch), 1026 (C–S). ^1^H NMR (DMSO-d_6_, 400 MHz) δ = 2.39 (s, 6H, 2CH_3_), 7.28 (d, 1H, *J* = 3.6 Hz, thienyl-C_3_/*H*), 7.94 (dd, 1H, thienyl-C_4_/*H*), 8.11 (d, d, 1H, *J* = 5.2 Hz, thienyl-C_5_/*H*), 7.55–7.77 (m, 10H, ArH’s), 10.75 (s, 2H, 2OH) ppm. ^13^C NMR (DMSO-d_6_) δ = 160.30, 149.81, 140.61, 137.61, 128.20, 127.81, 127.70, 127.52, 126.62, 122.80, 114.11, 34.91, 13.70 ppm. Anal. Calcd for C_25_H_22_N_4_O_2_S (442.54): C, 67.85; H, 5.06; N, 12.66; S, 7.24. Found; C, 67.78; H, 5.01; N, 12.56; S, 7.14%

### N-(3,5-Dimethyl-1,7-diphenyl-4-(thiophen-3-yl)-7,7a-dihydrodipyrazolo[3,4-b:4′,3′-e] pyridin-8(1H)-yl)-3,5-dimethyl-1,7-diphenyl-4-(thiophen-3-yl)-7,7dihydrodipyrazolo[3,4-b:4′,3′-e]pyridine-8(1H)-carbothioamid (14)

A mixture of pyrazole **13** (0. 02 mol) and thiosemicarbazide (0.01 mol) in sodium metal (0.02 g) dissolved in ethanol (20 ml) was heated under reflux for 6 h. The reaction mixture was cooled, and the separated solid product was filtered off, dried, crystallized from ethanol, and given compound **14** as brown crystals in 67% yield, m.p.112 °C. IR (KBr, ν, cm^−1^): broadband 3410–3445 (NH stretch), 2923 (aliphatic CH), 1270 (C=S). ^1^H NMR (DMSO-d_6_, 400 MHz) δ = 2.43 (s, 6H, 2CH_3_), 2.49 (s, 6H, 2CH_3_), 7.28 (d, 1H, *J* = 3.6 Hz, thienyl-C_3_/*H*), 7.94 (dd, 1H, thienyl-C_4_/*H*), 8.11 (d, d, 1H, *J* = 5.2 Hz, thienyl-C_5_/*H*), 715–7.79 (m, 20H, ArH’s), 10.80 (s, H, NH exchangeable by D_2_O) ppm. ^13^C NMR (DMSO-d_6_) δ = 177.41, 157.11, 155.61, 147.90, 143.80, 141.00, 139.71, 137.51, 130.71, 130.21, 129.71, 126.21, 28.41, 16.51, 14.81 ppm. Anal. Calcd for C_51_H_41_N_11_S_3_ (904.15); C, 67.73; H, 4.571; N, 17.00; S, 10.59. Found: C, 67.75; H, 4.57; N, 17.04; S, 10.64%.

### 1-(3,5-Dimethyl-1,7-diphenyl-4-(thiophen-3-yl)-7,7a-dihydrodipyrazolo[3,4-b:4′,3′-e] pyridin-8(1H)-yl) urea (15)

A mixture of pyrazole **13** (0. 01 mol) and semicarbazide (0.01 mol) in sodium metal (0.02 g) dissolved in ethanol (20 ml) was heated under reflux for 6 h. The reaction mixture was cooled, and the separated solid product was filtered off, dried, crystallized from ethanol, and given compound **15** as brown crystals in 60% yield, m.p.160 °C. IR (KBr, ν, cm−^1^): broadband 3420–3500 (NH stretch), 2920 (aliphatic CH), 1679 (C=O amide), 1636 (C=N). ^1^H NMR (DMSO-d_6_, 400 MHz) δ = 2.42 (s, 3H, CH_3_), 2.49 (s, 3H, CH_3_), 3.35 (s, 1H, methane), ^1^H NMR (DMSO-d_6_, 400 MHz) δ = 2.39 (s, 6H, 2CH_3_), 7.28 (d, 1H, *J* = 3.6 Hz, thienyl-C_3_/*H*), 7.94 (dd, 1H, thienyl-C_4_/*H*), 8.11 (d, d, 1H, *J* = 5.2 Hz, thienyl-C_5_/*H*), 7.40–7.79 (m, 10H, ArH's), 9.84 (s, 2H, NH_2_ exchangeable by D_2_O), 10.80 (s, H, NH exchangeable by D_2_O) ppm. ^13^C NMR δ = 162.78 (C=O), 161.87 157.82, 149.92, 146.32, 144.10, 136.67, 133.90, 129.56, 127.21, 120.86, 114.29, 113.23, 100.47, 98.37, 39.50, 21.98, 14.89 ppm. Anal. Calcd for C_26_H_23_N_7_OS (481.58); C, 64.90; H, 4.81; N, 20.36; S, 6.76. Found: C, 64.85; H, 4.75; N, 20.30; S, 6.66%.

### 3,5-Dimethyl-1,7-diphenyl-4-(thiophen-3-yl)-1,7,7a,8-tetrahydrodipyrazolo-[3,4-b:4′,3′-e] pyridine (16)

A mixture of pyrazole **13** (0. 01 mol) and ammonium acetate (0.01) in acetic acid (30 ml) in acetic acid (30 ml) was heated under reflux for 6 h. The reaction mixture was cooled, and the separated solid product was filtered off, dried, crystallized from ethanol, and given compound **16** as brown crystals in 60% yield, m.p.102 °C. IR (KBr, ν, cm^−1^): broadband 3370–3520 (NH stretch), 3069 (CH aromatic), 2921 (CH aliphatic), 1677 (C=N stretch), 1207 (C–S stretch). ^1^H NMR (DMSO-d_6_, 400 MHz) δ = 2.41(s, 3H, CH_3_), 2.43 (s, 3H, CH_3_ ), ^1^H NMR (DMSO-d_6_, 400 MHz) δ = 2.39 (s, 6H, 2CH_3_), 7.28 (d, 1H, *J* = 3.6 Hz, thienyl-C_3_/*H*), 7.94 (dd, 1H, thienyl-C_4_/*H*), 8.11 (d, d, 1H, *J* = 5.2 Hz, thienyl-C_5_/*H*), 7.37–7.78 (m, 10H, ArH’s), 10.78 (s, H, NH exchangeable by D_2_O) ppm. ^13^C NMR δ = 162.87, 161.82, 158.93, 154.15, 149.93, 149.88, 146.21, 144.47, 144.05, 136.50(C=N), 129.53, 127.21, 120.76, 120.58, 114.23, 113.19, 104.48, 101.96, 100.37, 98.34, 39.50, 21.95, 19.15, 14.84, 14.27 ppm. Anal. Calcd for C_25_H_21_N_5_S (423.54); C, 70.95; H, 5.15; N, 16.54; S, 7.62. Found: C, 70.90; H, 5.00; N, 16.49; S, 7.57%.

### 3,5-Dimethyl-1,7-diphenyl-4-(thiophen-3-yl)-1,7,7a,8-tetrahydropyrazolo[4,3-f] indazole-8-carboxylicacid (19)

A mixture of pyrazole **13** (0. 01 mol) and ethyl acetoacetate (0.01 mol) in ethanol (20 ml) and triethylamine (5 drops) was heated under reflux for 6 h. The reaction mixture was cooled, and the separated solid product was filtered off, dried, crystallized from ethanol, and given compound **19** as brown crystals in 60% yield, m.p.180 °C. IR (KBr, ν, cm^−1^): broadband 3420–3450 (OH), 2920 (aliphatic CH), 3069 (aromatic CH), 1677 (C=O carboxylic), 1270(C=S). ^1^H NMR (DMSO-d_6_, 400 MHz) δ = 2.41 (s, 6H, 2CH_3_), 3.33 (s, 1H, = CH), 7.28 (d, 1H, *J* = 3.6 Hz, thienyl-C_3_/*H*), 7.94 (dd, 1H, thienyl-C_4_/*H*), 8.11 (d, d, 1H, *J* = 5.2 Hz, thienyl-C_5_/*H*), 7.40–7.78 (m, 10H, ArH’s), 10.78 (s, H, OH exchangeable by D_2_O) ppm. ^13^C NMR in CDCL3 δ = 164.11 (C=O), 162.79, 150.29, 146.03, 144.28, 136.95, 134.04, 129.27, 126.95, 120.60, 114.49, 113.07, 100.14, 98.83, 77.85, 77.46, 77.26, 76.94, 40.59, 40.39, 40.18, 39.97, 39.76, 39.55, 39.34, 31.79, 29.56, 29.22, 22.52, 15.10, 14.03 ppm. Anal. Calcd for C_27_H_22_N_4_O_2_S (466.56); C, 69.59; H, 4.75; N, 12.10; S, 6.95. Found: C, 69.51; H, 4.69; N, 12.01; S, 6.87%.

### 3,6-Dimethyl-1-phenylpyrano[2,3-c] pyrazol-4(1H)-one (21)

A mixture of pyrazole **12** (0. 01 mol) and acetic anhydride (0.01 mol) in dioxane (20 ml) was heated under reflux for 4 h. The reaction mixture was cooled, and the separated solid product was filtered off, dried, crystallized from ethanol, and given compound **21** as pale-yellow crystals in 67% yield, m.p.220 °C. IR (KBr, ν, cm^−1^): 3090 (aromatic CH), 2924 (aliphatic CH), 1676 (C=O ketone). ^1^H NMR (DMSO-d6, 400 MHz) δ = 2.30 (s, 3H, CH_3_), 2.49 (s, 3H, CH_3_), 3.34 (s, H, ethylene), 7.34–7.92 (m, 5H, ArH’s) ppm. ^13^C NMR (DMSO-d_6_) δ = 175.61, 158.41, 153.90, 148.11, 137.61, 128.21, 127.81, 122.81, 105.51, 101.11, 19.90, 13.71 ppm. Anal. Calcd for C_14_H_12_N_2_O_2_ (240.26); C, 70.04; H, 5.10; N, 11.76. Found: C, 69.99; H, 5.03; N, 11.66%.

### 2-(3-Methyl-5-oxo-1-phenyl-4,5-dihydro-1H-pyrazole-4-carbonyl) benzoicacid (22)

A mixture of pyrazole **12** (0. 01 mol) and phthalic anhydride (0.01 mol) in dioxane (20 ml) was heated under reflux for 4 h. The reaction mixture was cooled, and the separated solid product was filtered off, dried, crystallized from ethanol, and given compound **22** as yellow crystals in 62% yield, m.p. 116 °C. IR (KBr, ν, cm^−1^): broadband 3350–3500(OH stretching for a carboxylic group), 3070 (aromatic CH), 2922 (aliphatic CH), 1756 (C=O ketone), 1677 (C=O carboxylic), 1634 (C=O). ^1^H NMR (DMSO-d_6_, 400 MHz) δ = 2.43 (s, 3H, CH_3_), 3.33(s, 1H, = CH), 7.40–7.78 (m, 9H, ArH’s), 10.77 (s, H, OH exchangeable by D_2_O) ppm. ^13^C NMR δ = 162.84 (C=O), 161.81, 158.88, 154.06, 149.91, 149.83, 146.11, 144.4, 144.01, 136.49, 129.48, 127.14, 120.66, 120.47, 114.18 , 113.11, 104.45, 101.92, 100.25, 98.29, 39.50, 21.94, 19.11, 14.78, 14.23 ppm. Anal. Calcd for C_18_H_14_N_2_O_4_ (322.32); C, 67.13; H, 4.45; N, 8.69. Found: C, 67.08; H, 4.38; N, 8.60%.

### 4-(3-Methyl-5-oxo-1-phenyl-4,5-dihydro-1H-pyrazol-4-yl)-4-oxobutanoicacid (23)

A mixture of pyrazole **12** (0. 01 mol) and succinic anhydride (0.01 mol) in dioxane (20 ml ) was heated under reflux for 4 h. The reaction mixture was cooled, and the separated solid product was filtered off, dried, crystallized from ethanol, and given compound **23** as yellow crystals in 64% yield, m.p.140 °C. IR (KBr, ν, cm^−1^): broadband 3430–3520 (OH stretch), 2921 (CH aliphatic), 1756 (C=O ketone), 1680 (C=O carboxylic). ^1^H NMR (DMSO-d_6_, 400 MHz) δ = 2.39 (s, 3H, CH_3_), 2.49 (s, 2H, CH_2_), 2.54 (s, 2H, CH_2_), 3.60 (s, 1H, CH=CH), 7.37–7.79 (m, 5H, ArH’s), 10.79 (s, H, OH exchangeable by D_2_O) ppm. ^13^C NMR (DMSO-d_6_) δ = 211.61, 177.81, 158.80, 152.52, 138.00, 128.20, 127.82, 122.81, 99.20, 38.40, 27.81, 16.00 ppm. Anal. Calcd for C_14_H_14_N_2_O_4_ (274.28); C, 61.39; H, 5.19; N, 10.21. Found: C, 61.31; H, 5.00; N, 10.12%.

### 3-(3-Methyl-5-oxo-1-phenyl-4,5-dihydro-1H-pyrazol-4-yl)-3-phenylpropanoic acid (24)

A mixture of pyrazole **12** (0. 01 mol) and cinnamic acid (0.01 mol) in dioxane (20 ml) was heated under reflux for 4 h. The reaction mixture was cooled, and the separated solid product was filtered off, dried, and crystallized from ethanol, giving compound **24** as white crystals in 59% yield, m.p.160 °C. IR (KBr, ν, cm^−1^): broadband 3435–3500 (OH stretch), 3067 (aromatic CH), 2922 (aliphatic CH), 1755 (C=O carboxylic), 1678 (C=O). ^1^H NMR (DMSO-d_6_, 400 MHz): δ = 2.42 (s, 3H, CH_3_), 2.57 (d, 2H, CH_2_), 3.33(s, 1H, methine), 7.39–7.77 (m, 10H, ArH’s), 10.78 (s, H, OH), ppm. ^13^C NMR (DMSO-d_6_) δ = 174.00, 170.11, 159.10, 139.80, 138.00, 128.41, 128.22, 127.81, 127.61, 122.80, 40.71, 37.81, 37.52, 16.00 ppm. Anal. Calcd for C_19_H_18_N_2_O_3_ (322.36); C, 70.85; H, 5.63; N, 8.74. Found: C, 70.79; H, 5.57; N, 8.69%.

### Antioxidant screening assay (ABTS method)

l-Ascorbic acid was obtained from Sigma, 2, 20-azinobis-(3-ethylbenzthiazoline-6-sulphonic acid) (ABTS) was purchased from Wak, and all other chemicals were of the highest quality available. For each of the investigated compounds, 2 ml of ABTS solution (60 mM) was added to 3 M magnesium oxide (MnO_2_) solution (25 mg/ml), all prepared in phosphate buffer (pH 7, 0.1 M). The mixture was shaken, centrifuged, and filtered, and the absorbance (A control) of the resulting green–blue solution (ABTS radical solution) was adjusted at ca. 0.5 at l 734 nm. Then, 50 ml of (2 mM) solution of the test compound in spectroscopic grade methanol/phosphate buffer (1:1) was added. The absorbance (A test) was measured, and the reduction in color intensity was expressed as % inhibition^26^. The Inhibition for each compound was calculated from the following equation.

$$ \% {\text{ Inhibition }} = \, \left[ {{\text{A }}\left( {{\text{control}}} \right) - {\text{A }}\left( {{\text{test}}} \right)/{\text{A }}\left( {{\text{Control}}} \right)} \right] \, \times { 1}00. $$

Ascorbic acid (vitamin C) was a standard antioxidant (positive control). A blank sample was run without ABTS and using methanol/phosphate buffer (1:1) instead of the sample. The negative control sample was run with methanol/phosphate buffer (1:1) instead of the tested compound.

### Antimicrobial activity

The antimicrobial activity of the tested compounds was determined using a modified Kirby‐Bauer disc diffusion method^33^. Briefly, 100 μl of the test bacteria/fungi were grown in 10 mL of fresh media until they reached a count of approximately 108 cells/ml for bacteria and 105 cells/ml for fungi^38^.

The antimicrobial activity of the synthesized compounds was tested against a panel of two-gram positive bacteria (*Staphylococcus aureus, Bacillus subtilis*) and two Gram-negative bacteria (*Escherichia coli, Pseudomonas aeruginosa*). In addition, the antifungal activities of the compounds were tested against two fungi (*Candida albicans and Aspergillus flavus*). Each of the compounds was dissolved in DMSO, and a solution of the concentration 1 mg/ml was prepared separately. Paper discs of Whitman filter paper were prepared with standard size (5 cm) and were cut and sterilized in an autoclave. The paper discs soaked in the desired concentration of the complex solution were placed aseptically in the Petri dishes containing nutrient agar media (agar 20g + beef extract 3g + peptone 5g) seeded with *Staphylococcus aureus, Bacillus subtilis, E. coli, Pseudomonas aeruginosa, Candida albicans*, and* Aspergillus flavus.* The Petri dishes were incubated at 36 c, and the inhibition zones were recorded after 24 h. Each treatment was replicated three times. The antibacterial activity of a common standard antibiotic, ampicillin, and antifungal Colitrimazole was also recorded using the same procedure as above at the same concentration and solvents. The formula calculated the % activity index for the complex as follows:

$$\% Activity \, Index= \frac{Zone \, of \, inhibition \, by \, test \, compound \, (diametre)}{Zone \, of \, inhibition \, by \, standard \, \left(diametre\right)}\times 100.$$

The other method of antimicrobial activity measurements was collecting the chemical subject samples under study in sterile and dry Eppendorf tubes with the specific information for each sample. Weight the under-study quantity of each sample and were dissolved in acetone, mixed for (2–5) min. Prepared 2 ml of each acetone dissolved subject in a Wizerman tube for each microbe under test and added the data on tube^39^. Collected the microbial identified isolates as pure and clear colonies, then made a microbial distilled water suspension of each as McFarland conc, with unique information settings^40^.

Applied a mixture of (acetone dissolved subject + microbial suspension), mixed the ingredients, and calculated the time. Cultured from each mixture every time recorded (1, 3, 5, and 7 h), done on suitable dish media with data recorded on each plate, and incubated at 35–37 °C for 24–48 h. Followed the results of microbial culture growth and recorded the number of colonies in each dish 36. Determined the percentage of microbial death by applying the Equation^41^:

$$ \left[ {\left( {{\text{Colony no}}./{3}00 \, \times { 1}00} \right) \, - {1}00} \right]. $$

Statistical analysis of the results and calculation of the arithmetic mean for each chemical material subject sample under study was shown in tables and graphs.

### Determination of minimal inhibitory concentration (MIC)

Stationary-phase bacteria cultures were prepared at 37°C and used to inoculate fresh ml culture to an OD600 of 0.05. The 5.0 ml cultures were then incubated at 37°C until an OD600 of 0.10 was achieved, from which standardized bacterial suspensions were prepared to a final cell density of 6 × 10^−5^ colony forming units (CFUs)/ml. Serial dilutions from the treatments (0–320 μg/ml) were prepared, mixed with 5.0 ml of the standardized bacteria suspension, added to the plates, and incubated for 24 h at 37 °C. The turbidity produced in each tube was recorded using a UV–visible spectrometer.

### Molecular docking

Molecular docking was analyzed via the CB-Dock server (http://clab.labshare.cn/cb-dock/php/index.php)^42,43^. CB-Dock2 automatically identifies binding sites, calculates center and size, customizes docking box size based on query ligands, and performs molecular docking with AutoDock Vina 1.1.2. For the target protein CCP, PDB ID: 2X08 (Resolution 2.01 Å) from the RCSB Protein Data Bank was selected. For the validation of the molecular docking study, 2X08’s cocrystal ligand ascorbic acid was re-docked, and the RMSD between the docking pose and the natural pose was measured as 0.712 Å. Visualization analysis of protein–ligand complexes was performed using UCSF Chimera v1.15 and BIOVIA Discovery Studio Visualizer v2021.

### Molecular dynamics simulations

The input files required for the MD simulation were created with the default settings of the Solution Builder on the CHARMM-GUI server (https://charmm-gui.org/). Topology files of proteins and compounds were created using Amber ff99SB force fields. MD simulation was performed using Gromacs version 2020.1. 1500 frames were recorded for the 150 ns MD simulation. RMSD and hydrogen bond analyses were performed using gmx rms, gmx hbond, gmx gyrate, and gmx rmsf scripts, respectively, and MD trajectory. RMSD and H bond plots were done with GraphPad Prism 8, and bond pose analysis and animation video rendering were done with UCSF Chimera v1.15^44,45^.

## Conclusion

In brief, the study presented a comprehensive assessment to describe **[3 + 3]** Cycloaddition of benzoyl isothiocyanate and pyrazolone **1**, which undergo oxidation cyclization producing pyrazolo oxadiazine **3**. The diol **5** was obtained as a condensation of two equivalents of **1** with thiophene-2-carboxaldehyde in acetic acid above the sodium acetate mixture. The starting compound is used to obtain polycyclic heterocyclic systems. In addition, chemical compounds positively influence antimicrobial and antioxidant approaches. Docking assays with cytochrome c peroxidase proteins [2X08] using ascorbic acid as the standard binder revealed that **13** and **16** have strong free radical-scavenging characteristics compared to ascorbic acid. POM theory proves the results of the biological studies by the Libnaiski rule, and finally, MD simulations at 150 ns validated the molecular docking results.

### Supplementary Information

Supplementary Figures.

Supplementary Video S1.

Supplementary Video S2.

## Data Availability

The datasets used and analyzed during the current study are available from the corresponding author upon reasonable request.

## Abbreviations

TEA: Trimethylamine
^1^HNMR: Nuclear magnetic resonance
IR: Infrared radiation
DMSO: Dimethyl sulfoxide
AC_2_O: Acetic anhydride
MD: Molecular dynamics
