# Supplementary material for: Synthesis of novel pyrazolone candidates with studying some biological activities and in-silico studies
Source: Sci Rep. 2023 Nov 6;13:19170. doi: 10.1038/s41598-023-43575-z (PMC10628256; doi:10.1038/s41598-023-43575-z)
Supplement: Supplementary file 1 — Supplementary Figures. [file 41598_2023_43575_MOESM1_ESM.pdf]

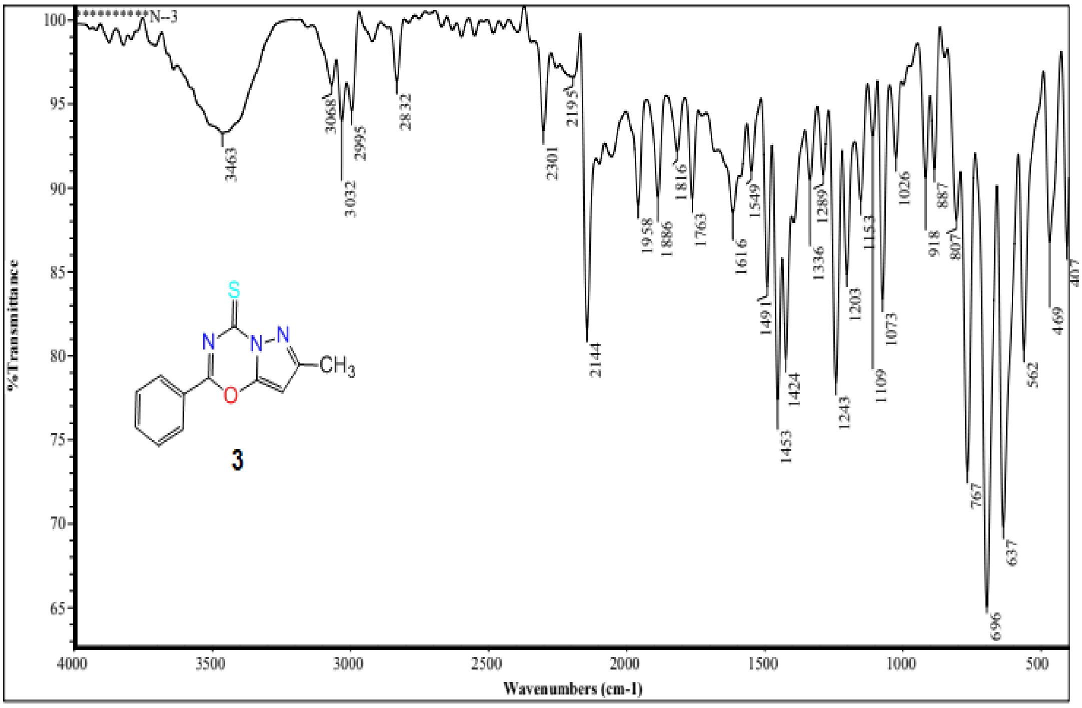

**********N--3

[illegible]

Figure S1: IR spectrum of compound **3**

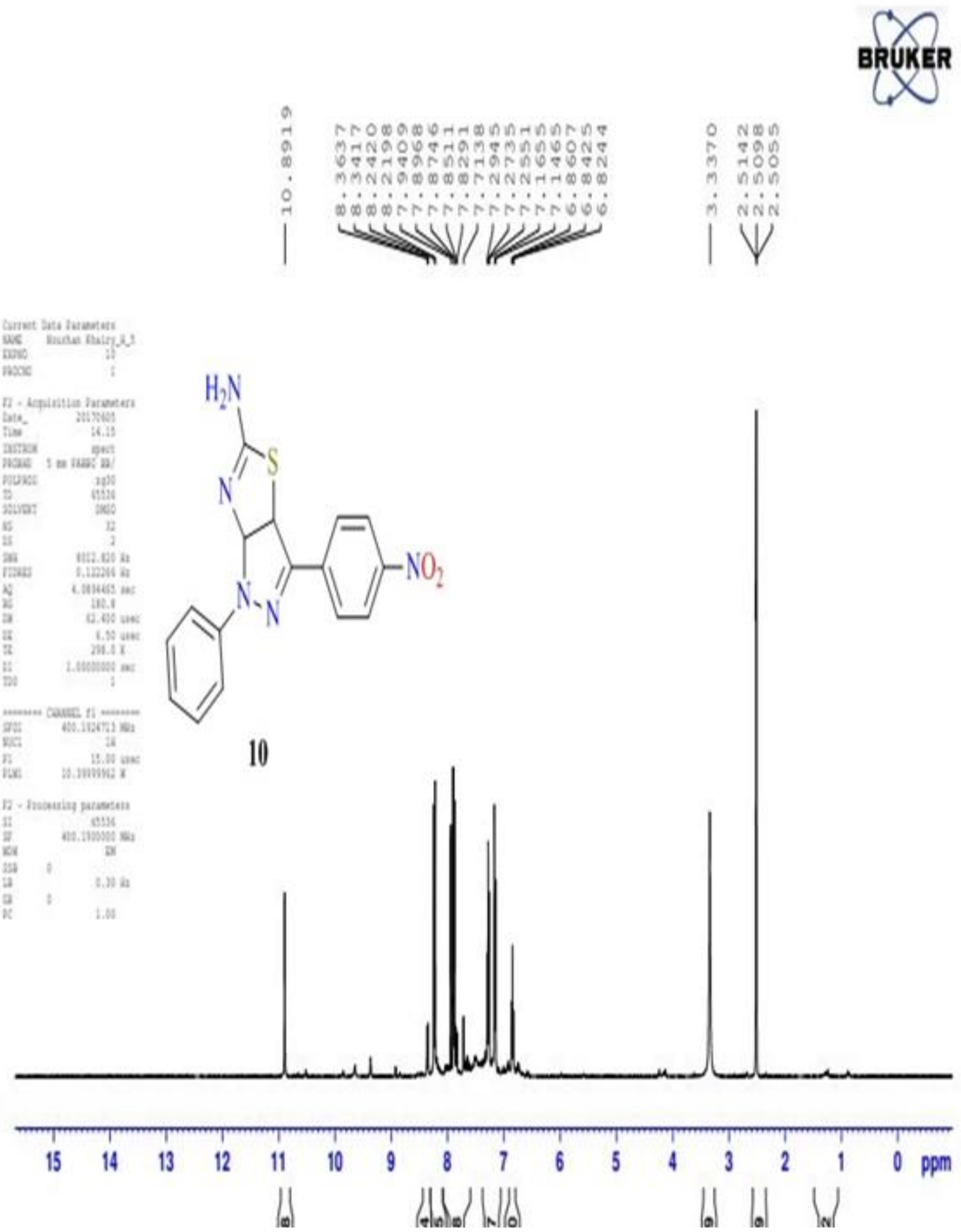

Figure S2: ^{1}H-NMR spectrum of compound **3**

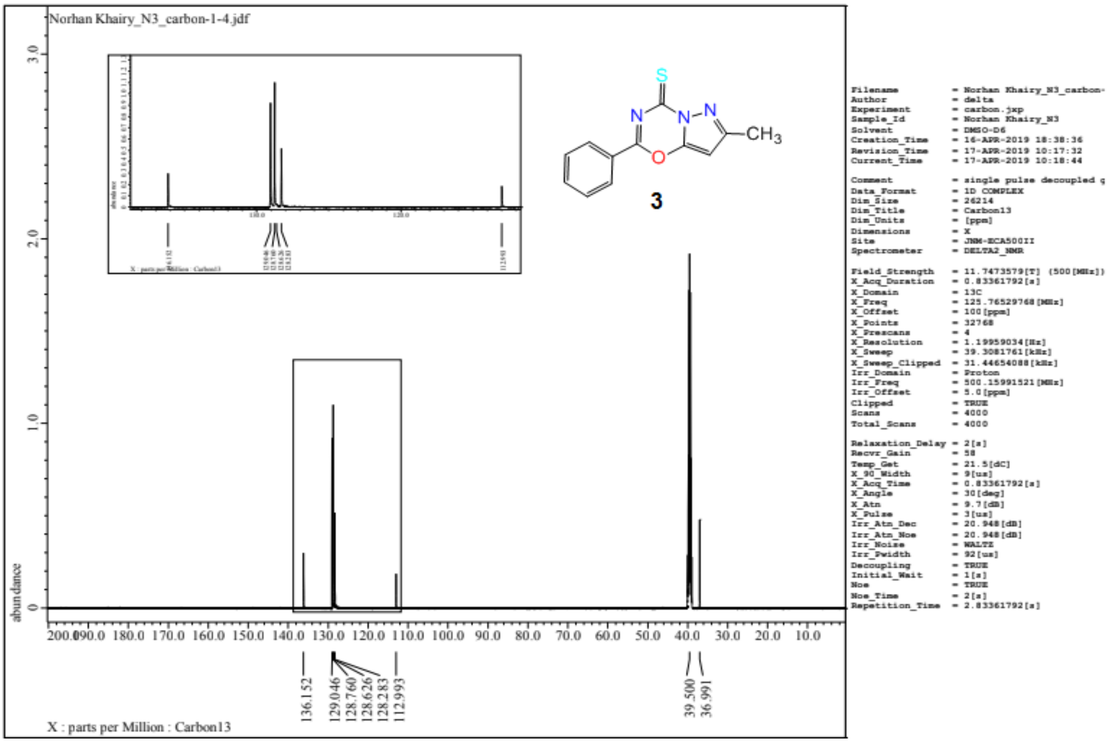

Figure S3: ^{13}C-NMR spectrum of compound **3**

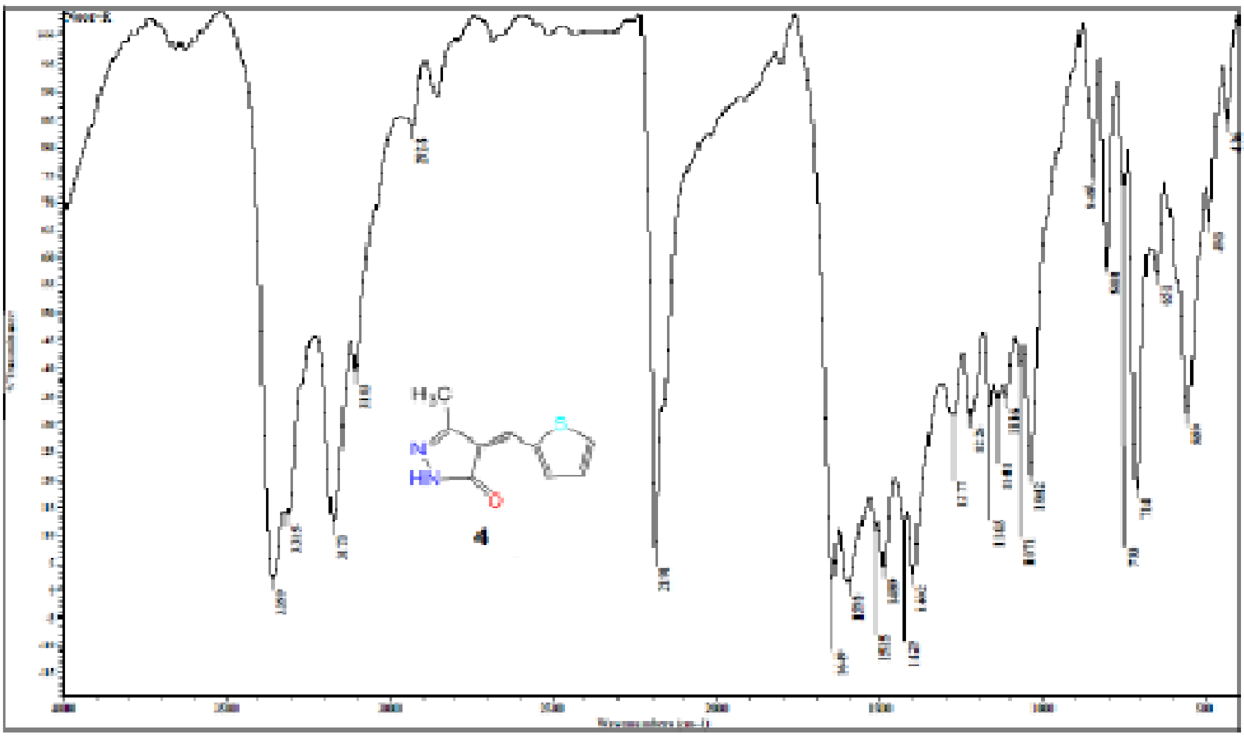

Figure S4: IR spectrum of compound **4**

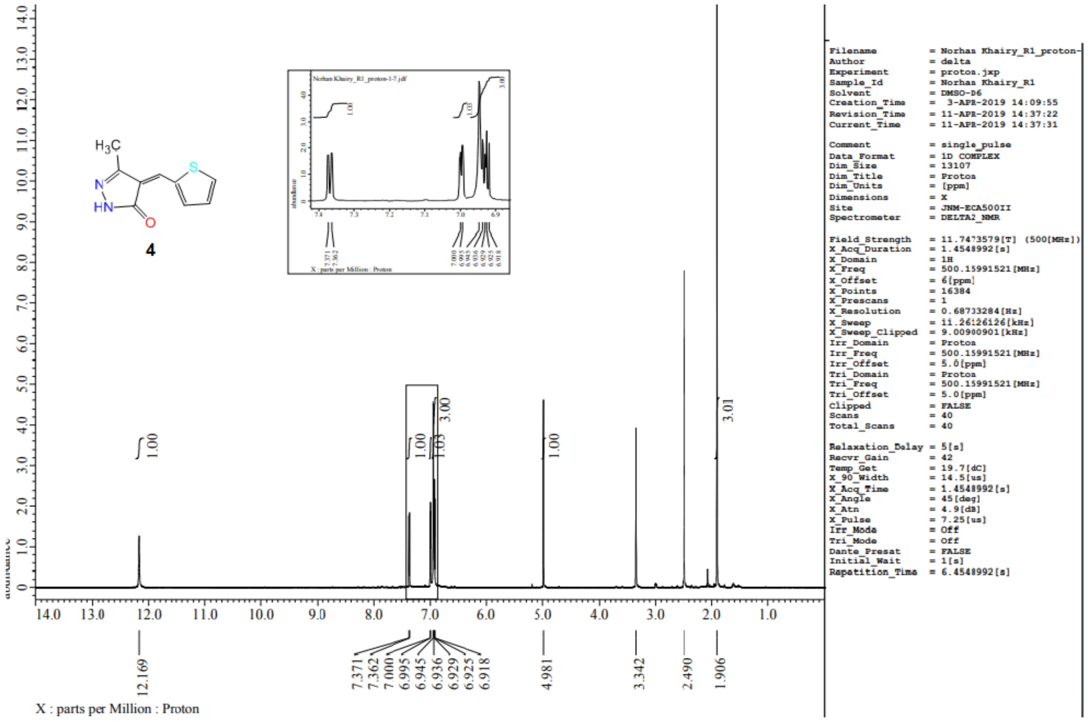

Figure S5: ^{1}H-NMR spectrum of compound **4**

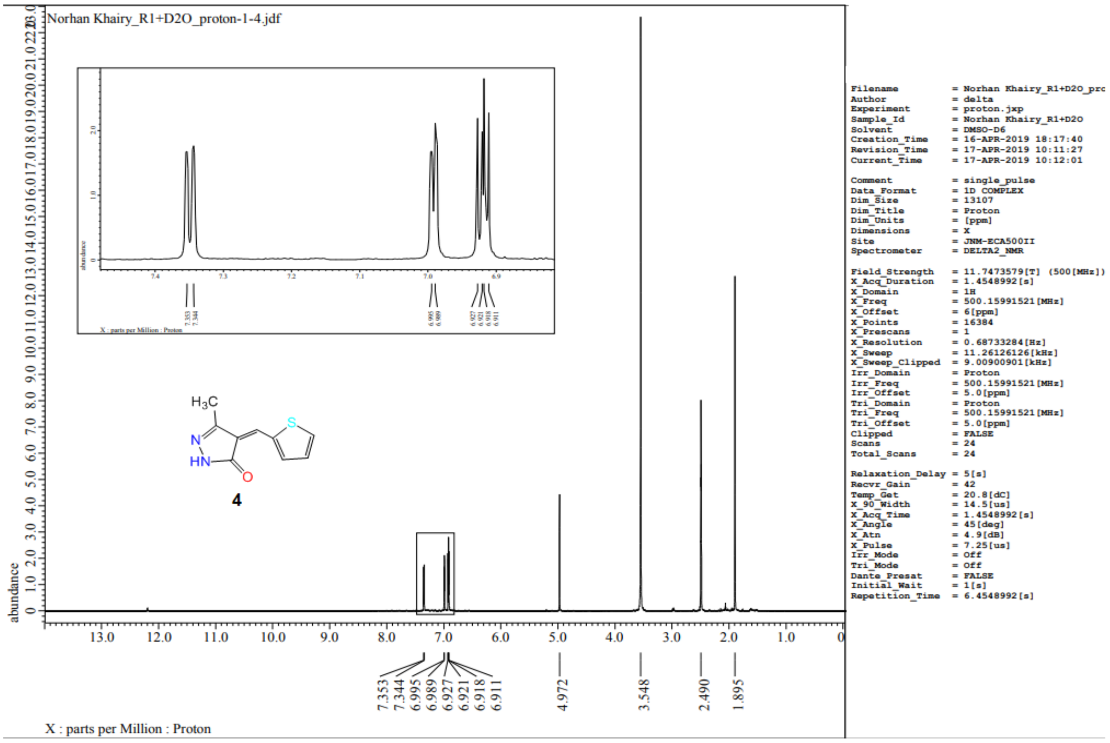

Figure S6: ^{1}H-NMR spectrum (D_2O) of compound **4**

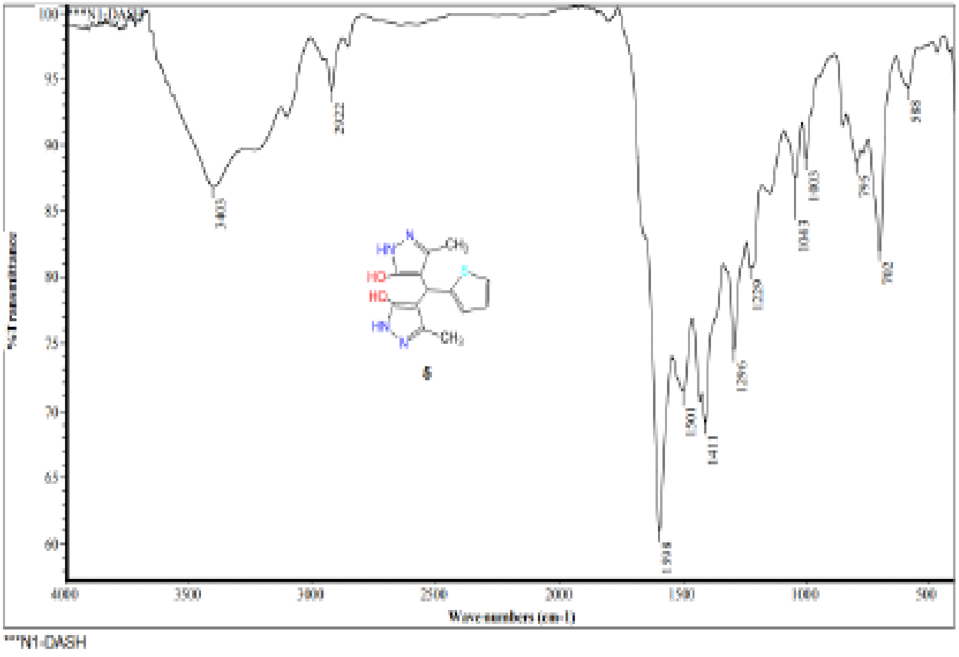

Figure S7: IR spectrum of compound **5**

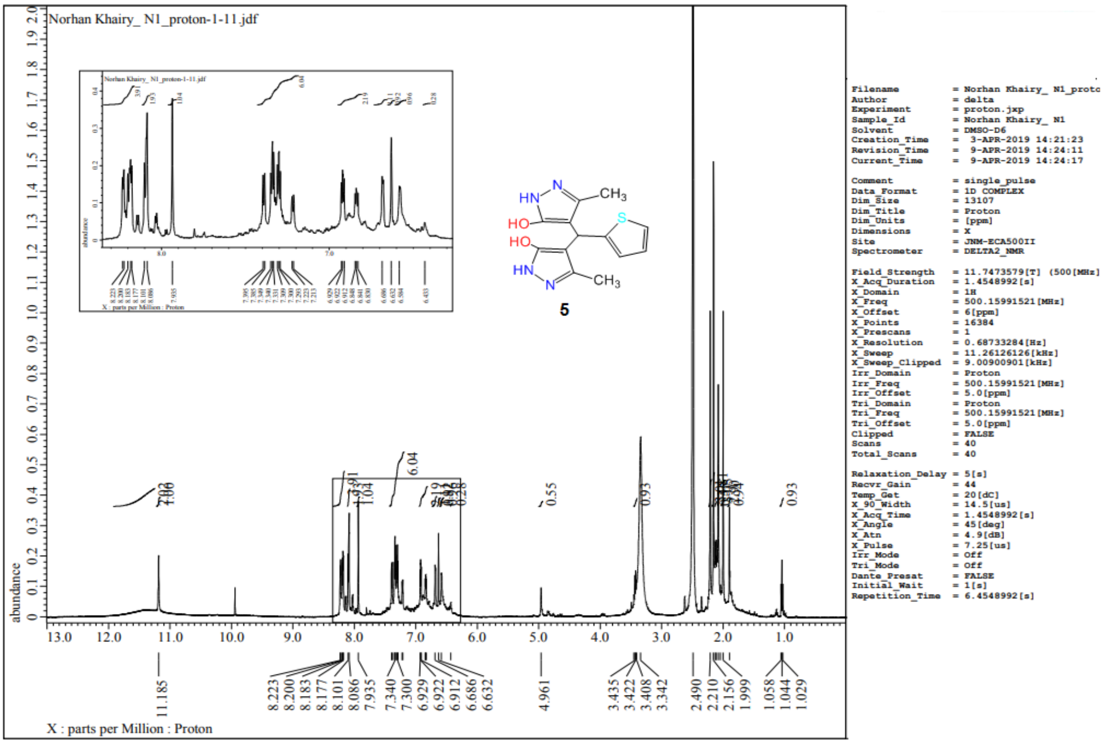

Figure S8: ^{1}H-NMR spectrum of compound **5**

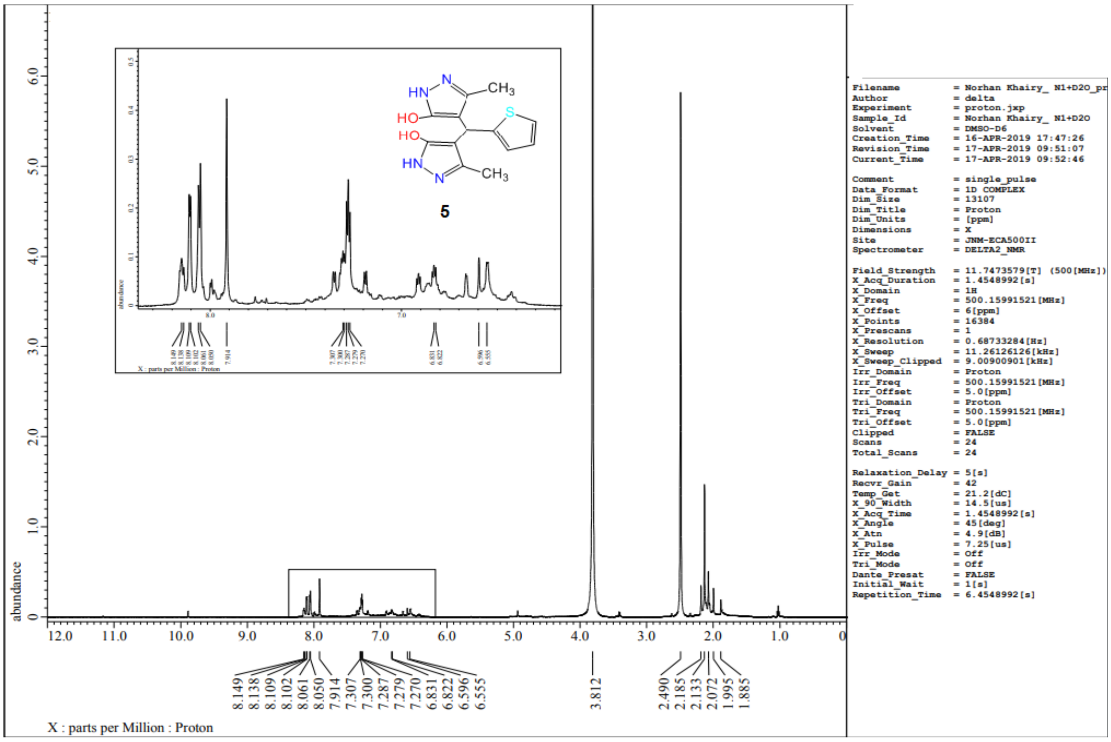

Figure S9: ^{1}H-NMR spectrum (D_2O) of compound **5**

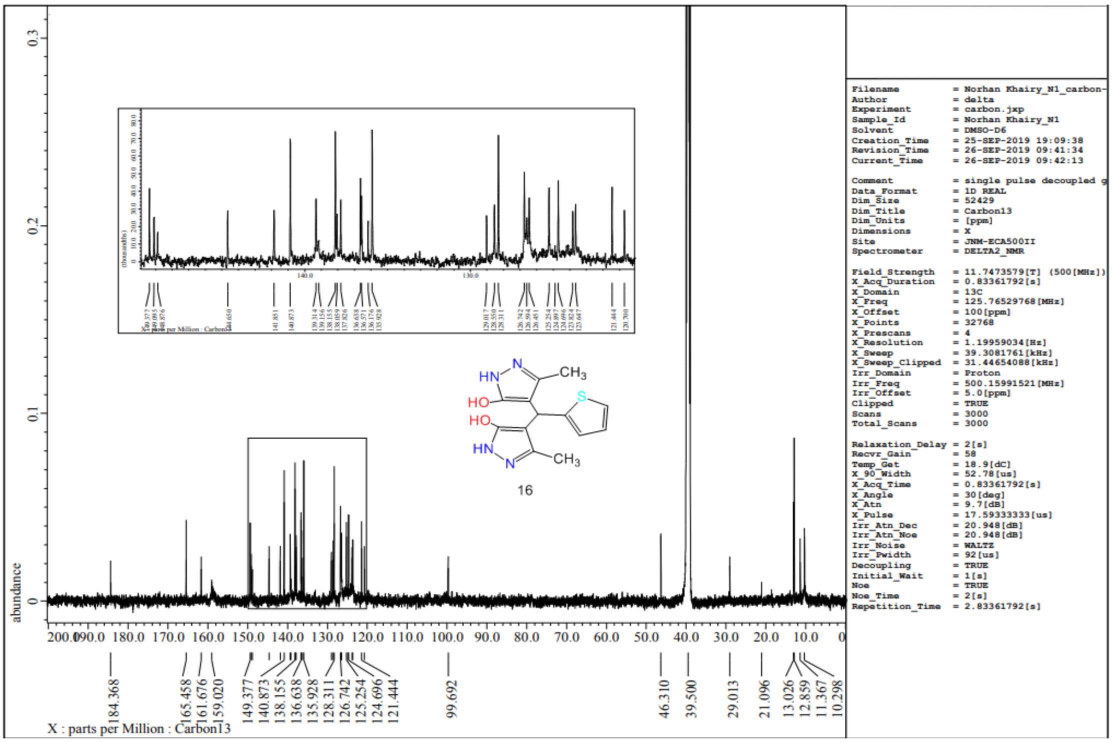

Figure S10: ^{13}C-NMR spectrum of compound **5**

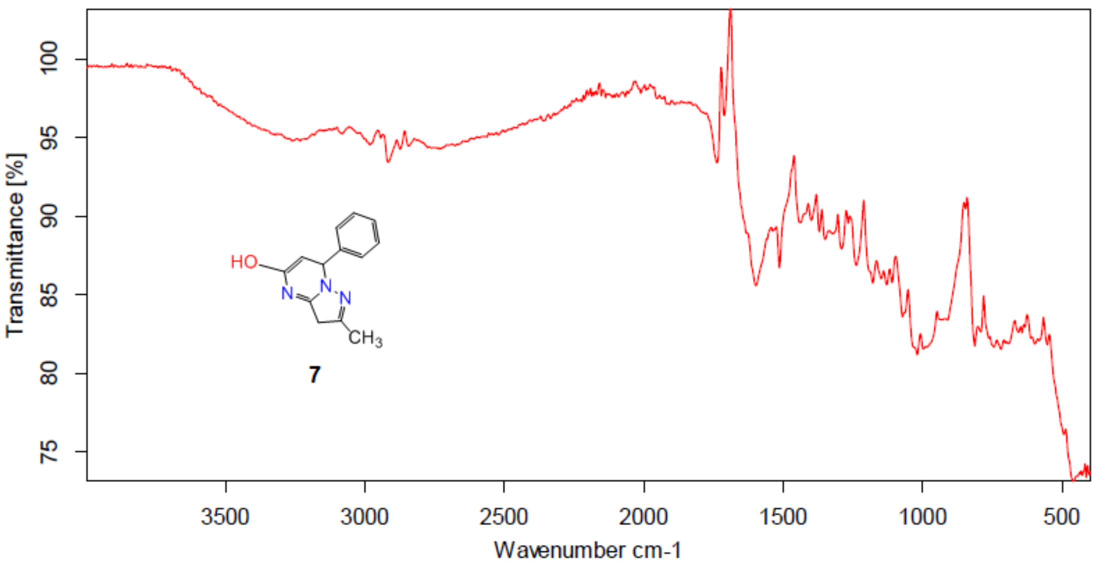

Figure S11: IR spectrum of compound **7**

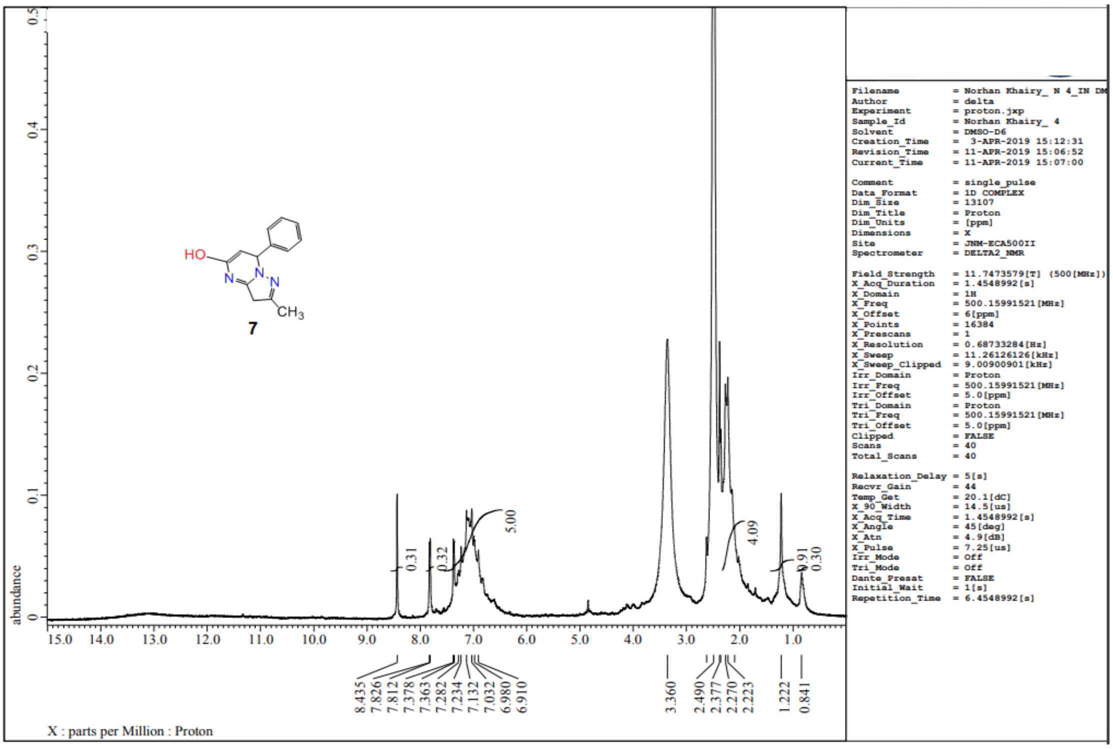

Figure S12: ^{1}H-NMR spectrum of compound **7**

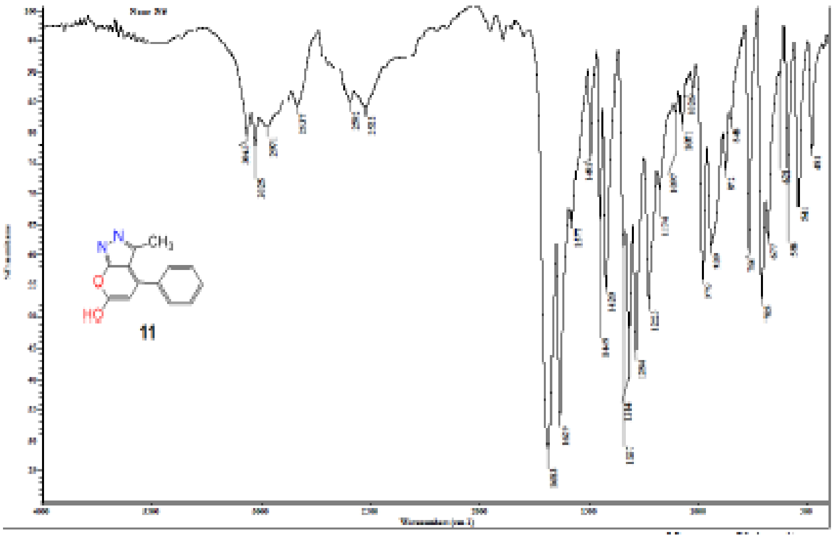

Figure S13: IR spectrum of compound **11**

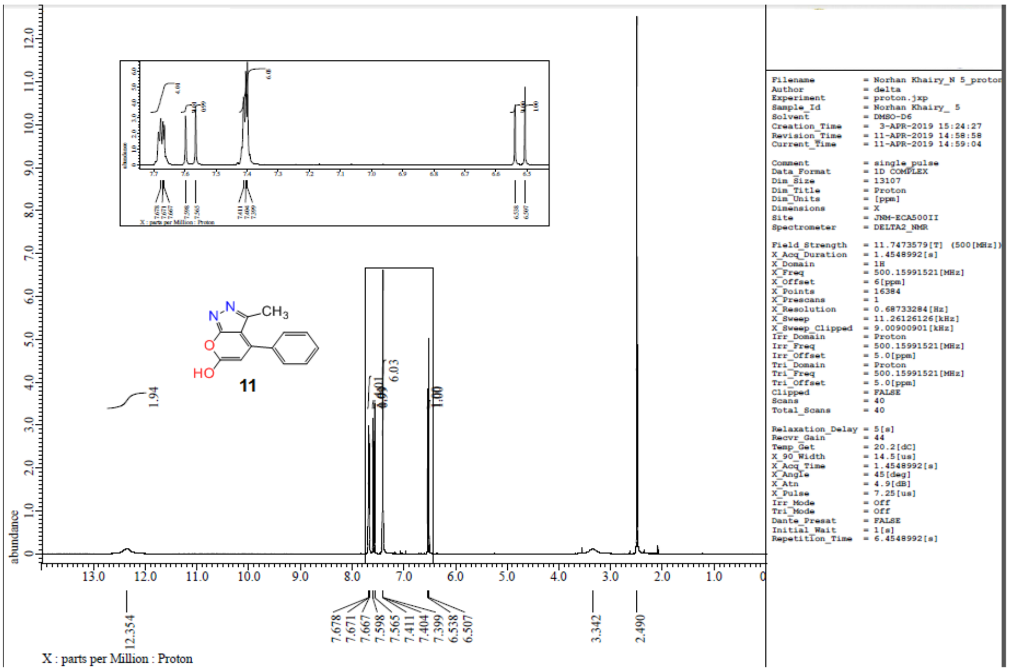

Figure S14: ^{1}H-NMR spectrum of compound **11**

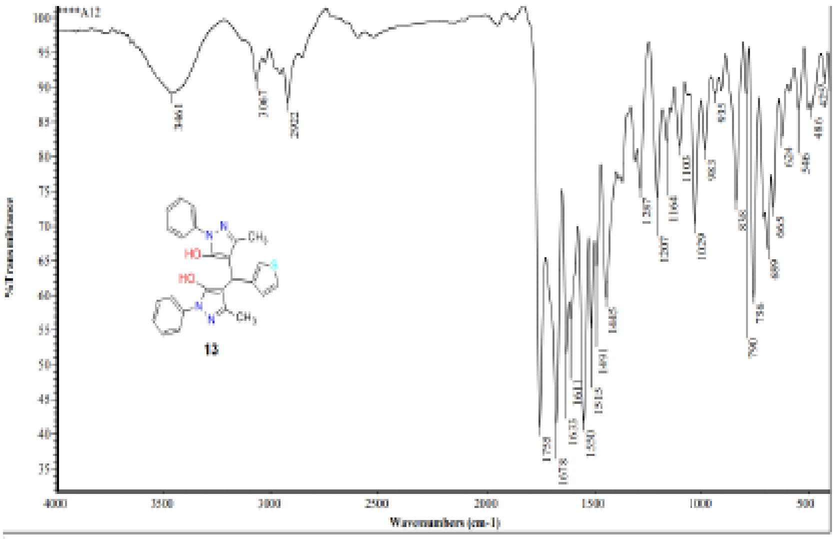

Figure S15: IR spectrum of compound **13**

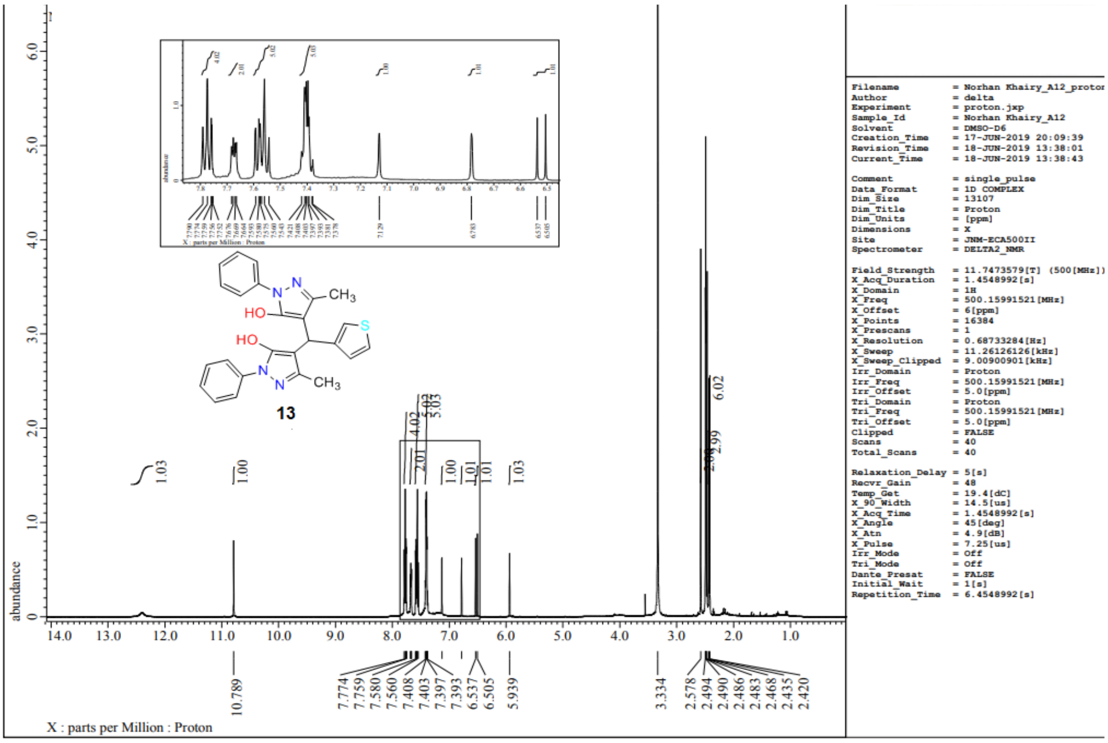

Figure S16: ^{1}H-NMR spectrum of compound **13**

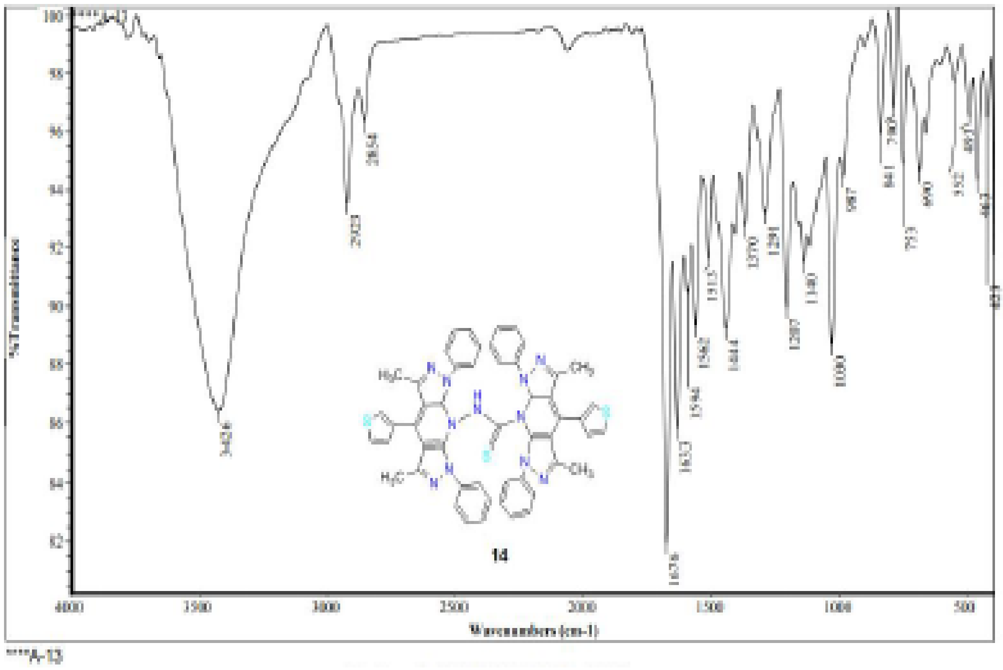

Figure S17: IR spectrum of compound **14**

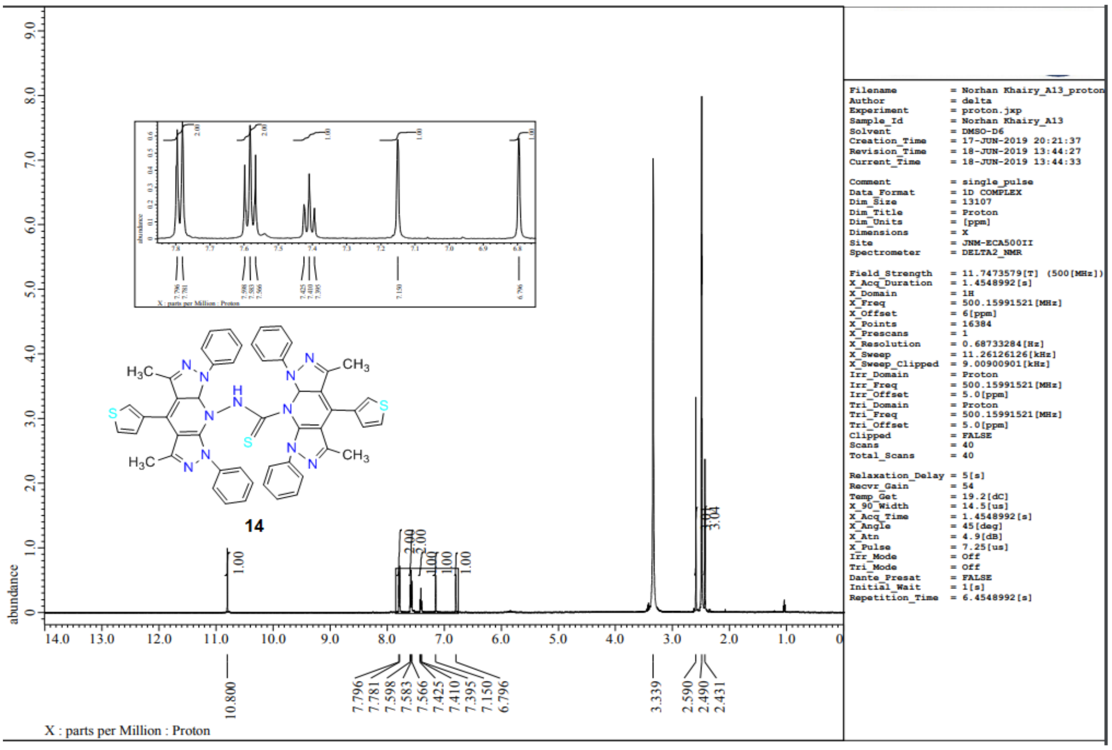

Figure S18: ^{1}H-NMR spectrum of compound **13**

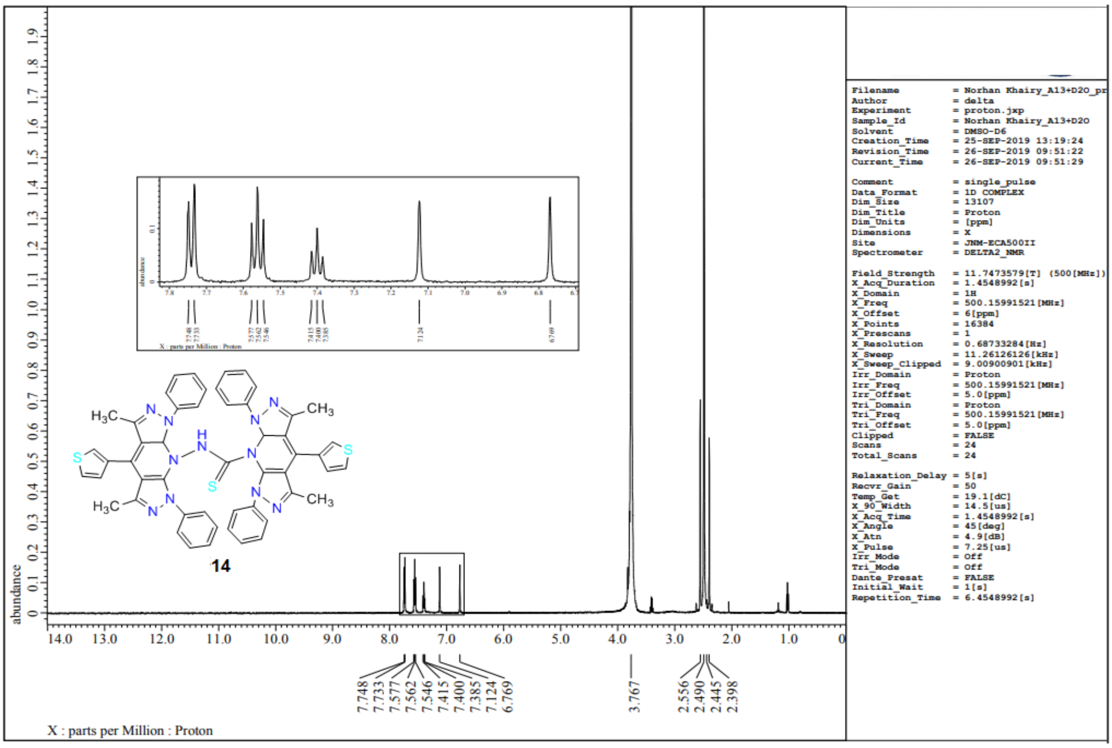

Figure S19: ^{1}H-NMR spectrum (D_2O) of compound **14**

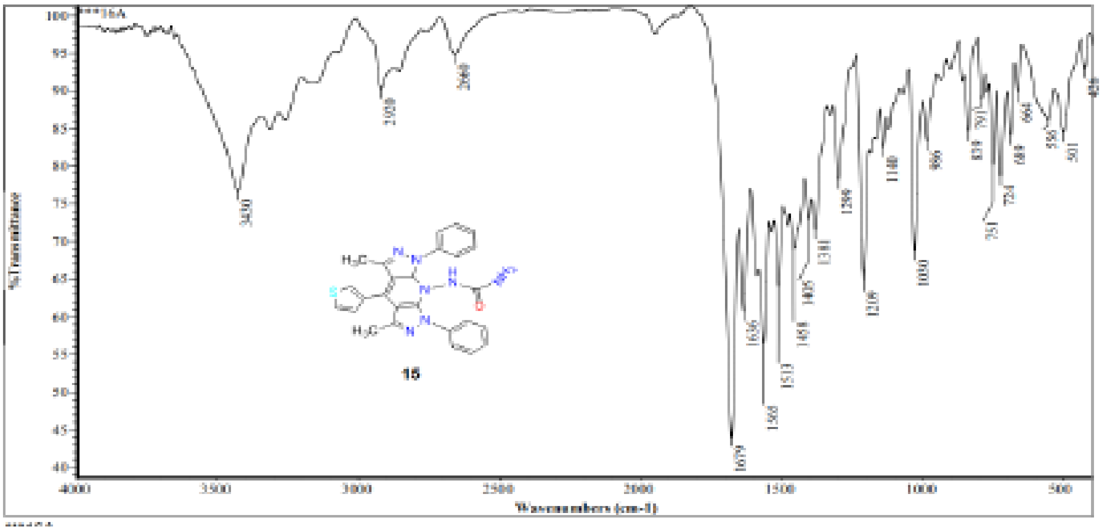

Figure S20: IR spectrum of compound **15**

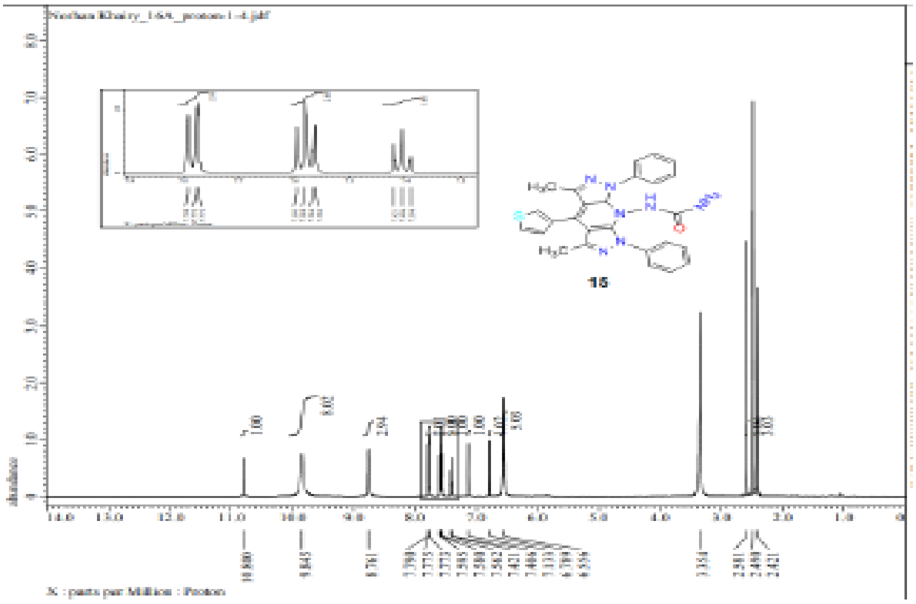

Figure S21: ^{1}H-NMR spectrum of compound **15**

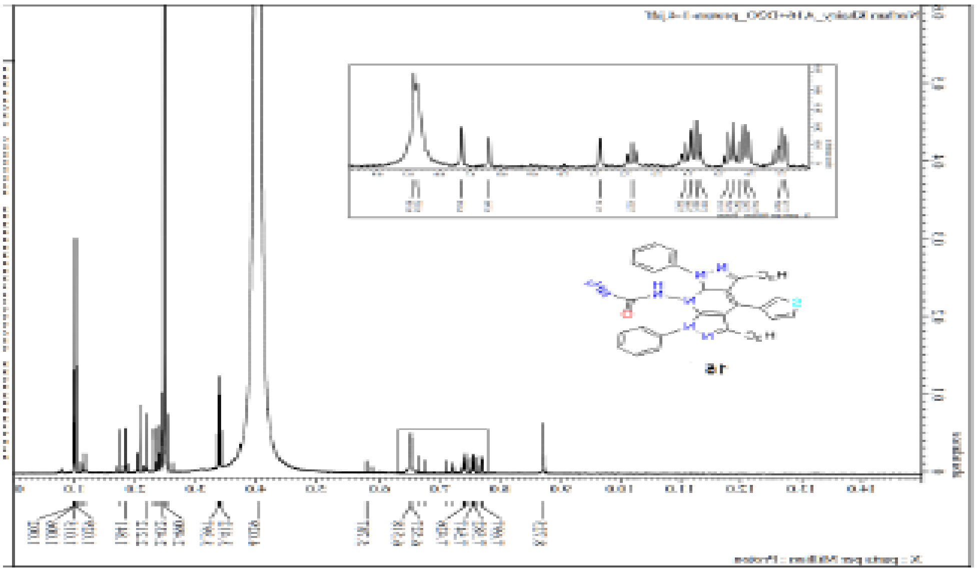

Figure S22: ^{1}H-NMR spectrum (D_2O) of compound **15**

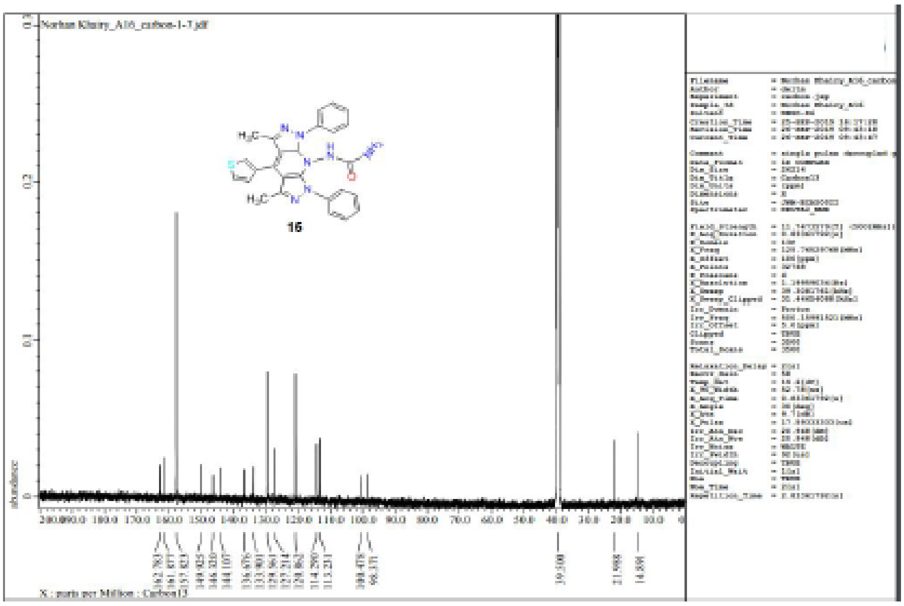

Figure S23: ^{13}C-NMR spectrum of compound **15**

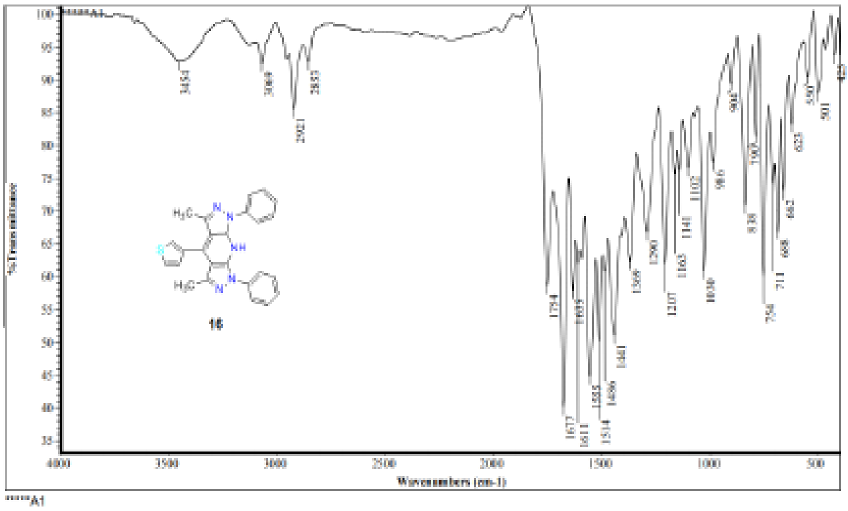

Figure S24: IR spectrum of compound **16**

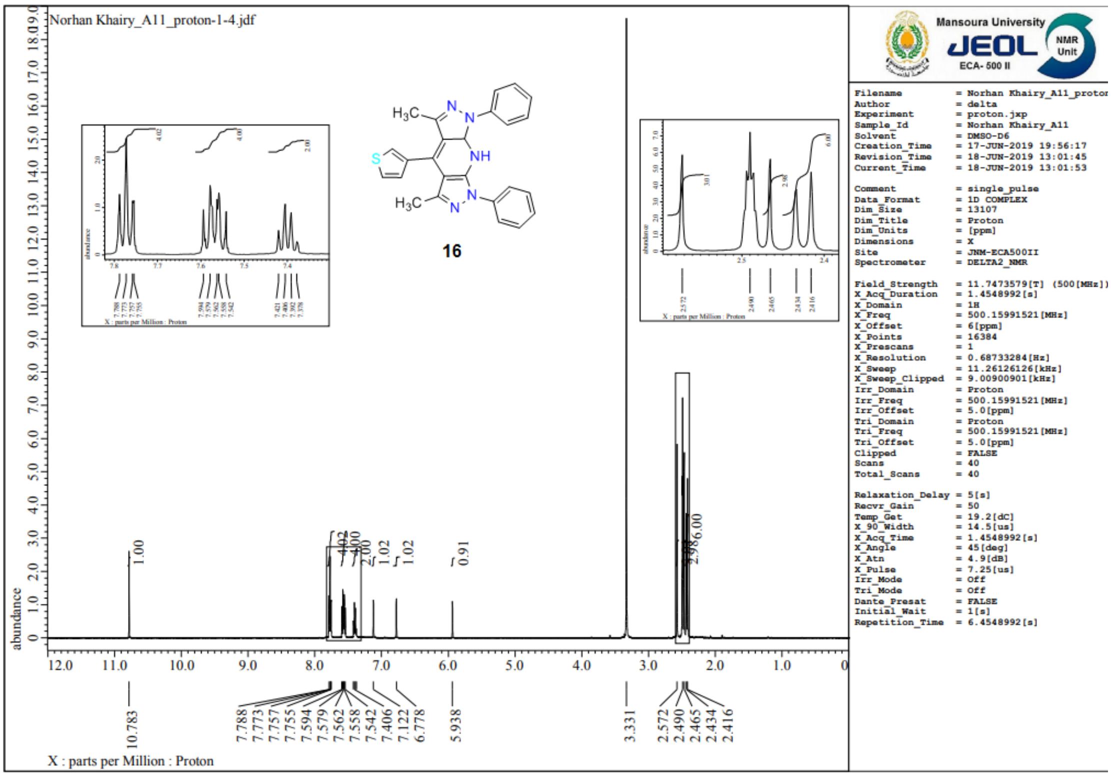

Figure S25: ^{1}H-NMR spectrum of compound **16**

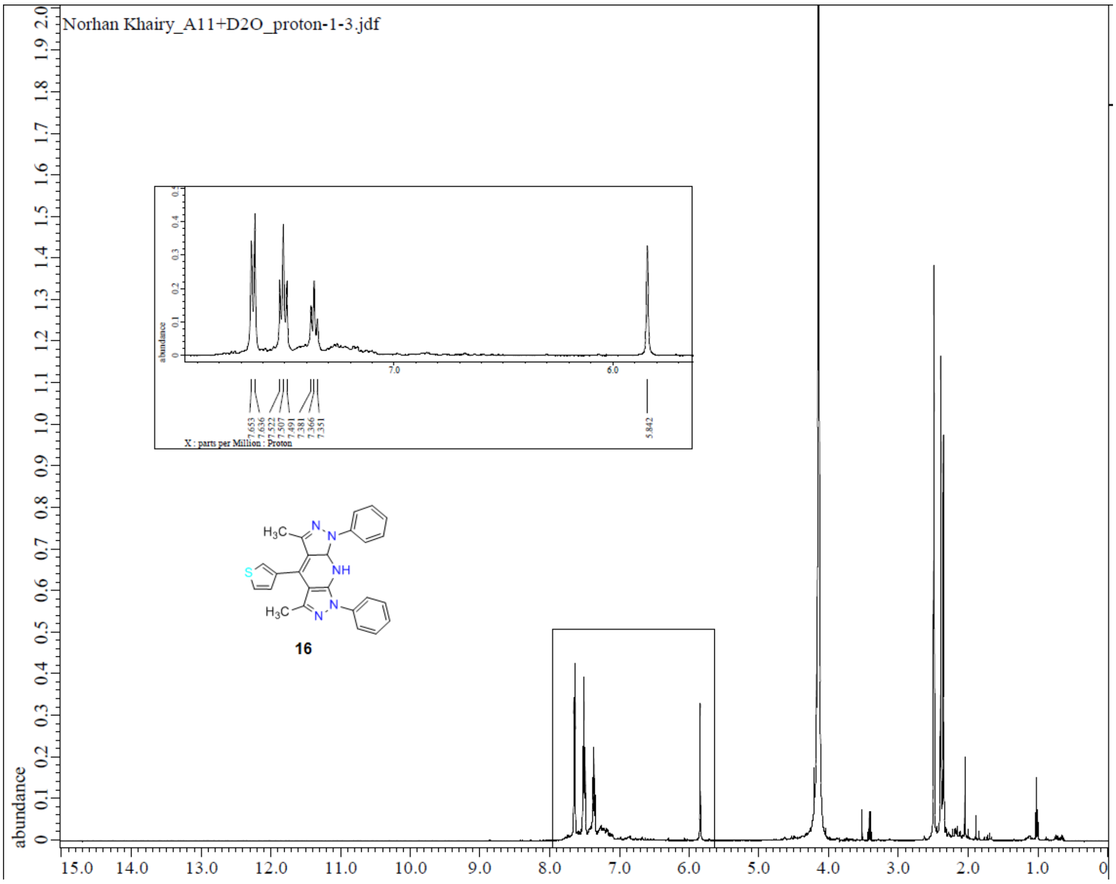

Figure S26: ^{1}H-NMR spectrum (D_2O) of compound **16**

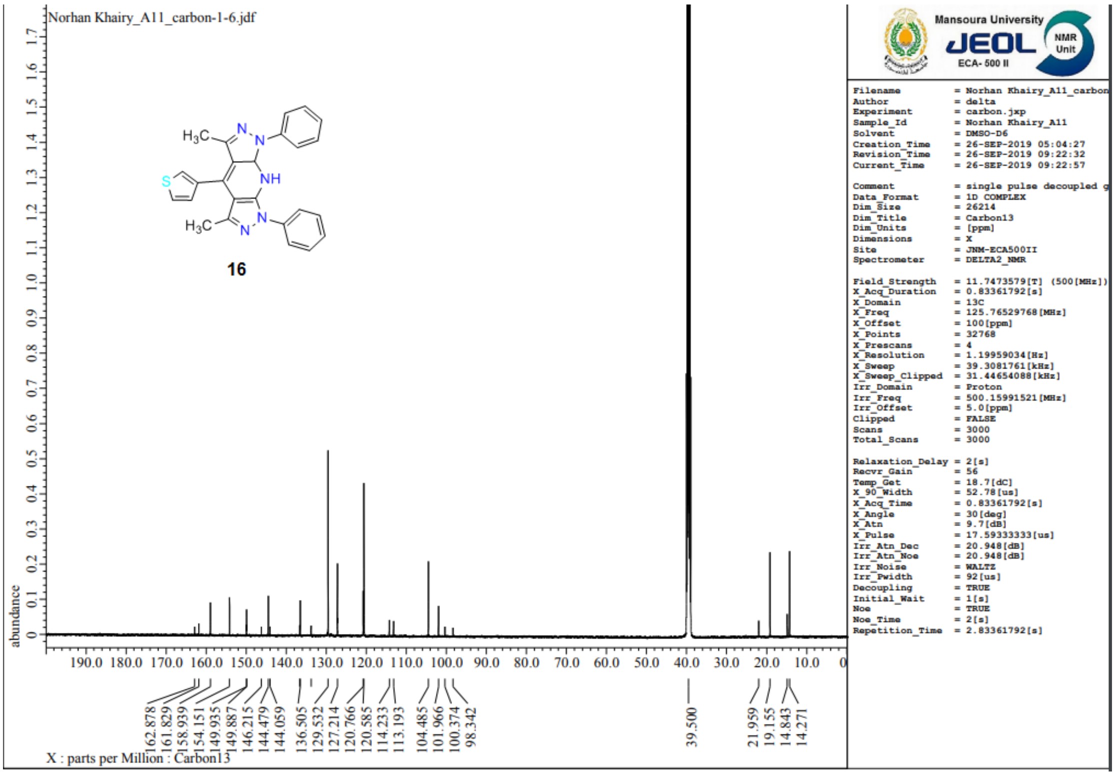

Figure S27: ^{13}C-NMR spectrum of compound **16**

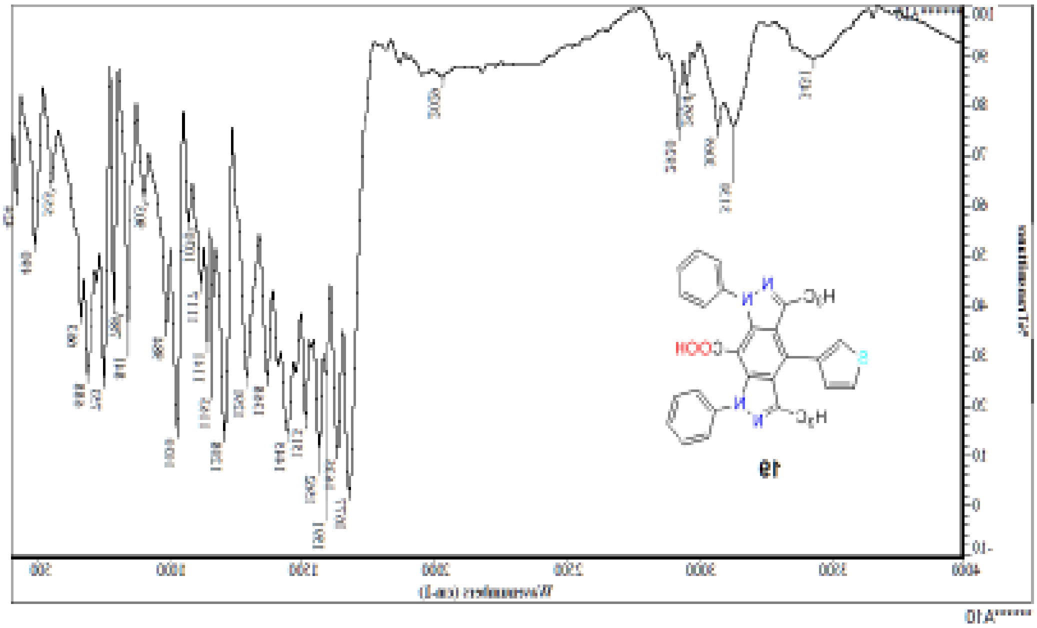

Figure S28: IR spectrum of compound **19**

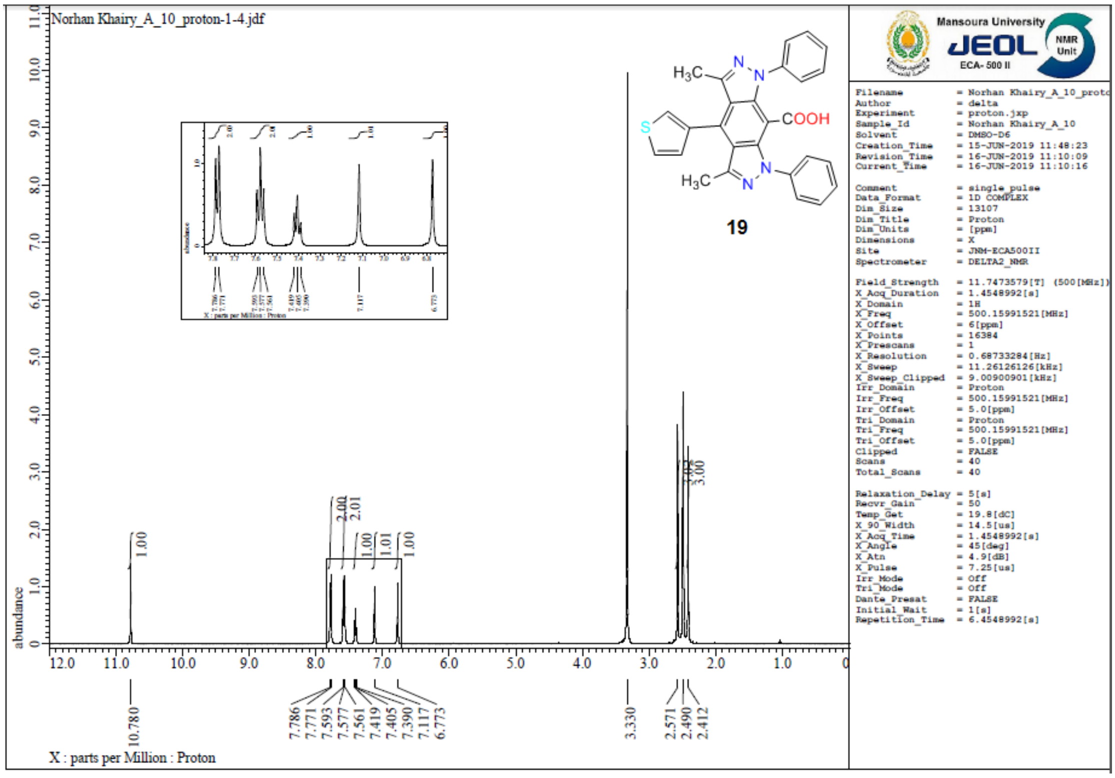

Figure S29: ^{1}H-NMR spectrum of compound **19**

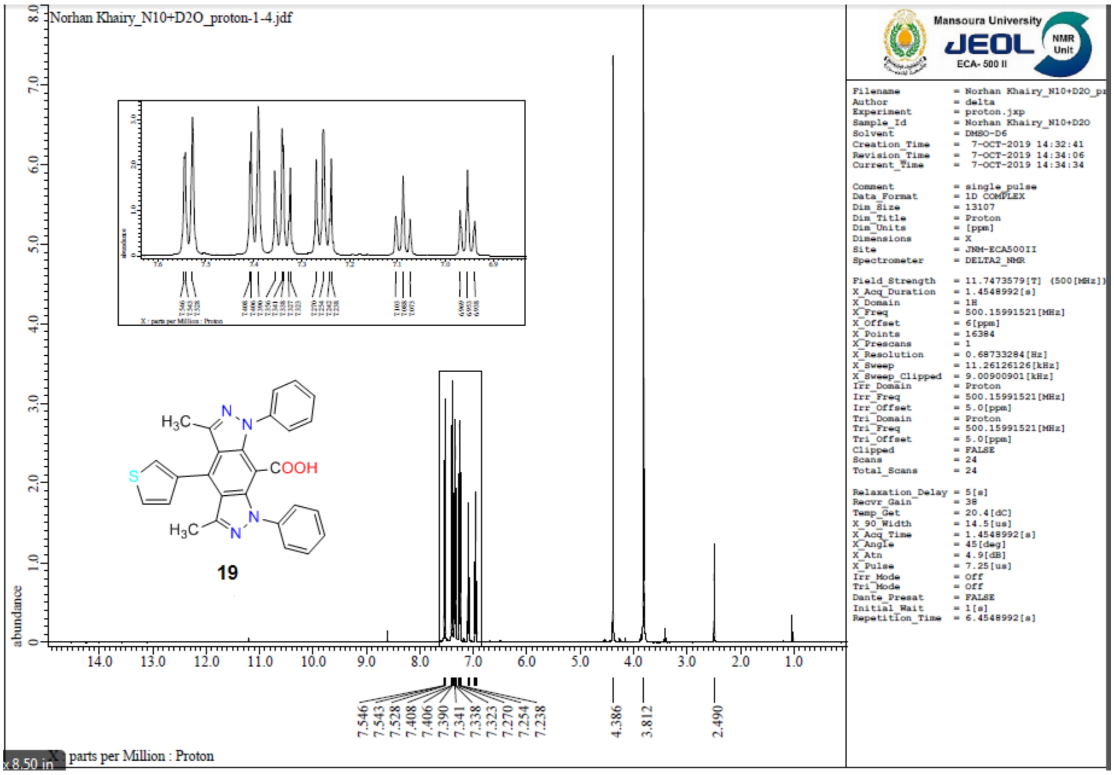

Figure S30: ^{1}H-NMR spectrum (D_2O) of compound **19**

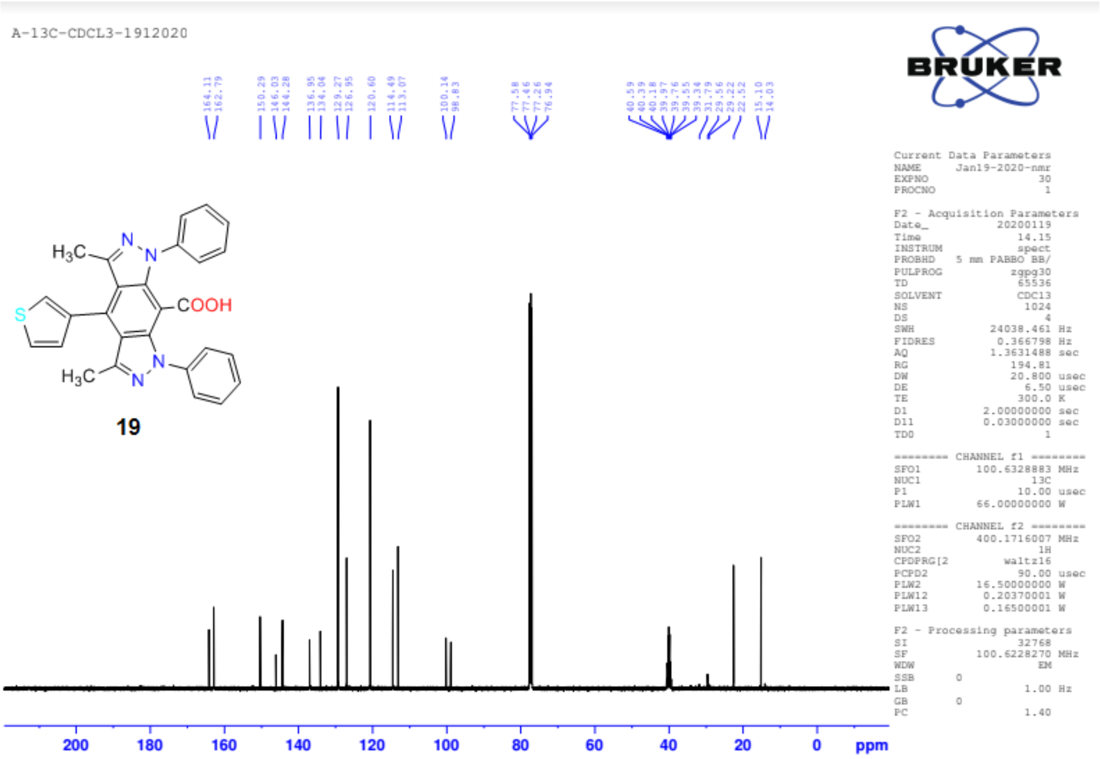

Figure S31: ^{13}C-NMR spectrum of compound **19**

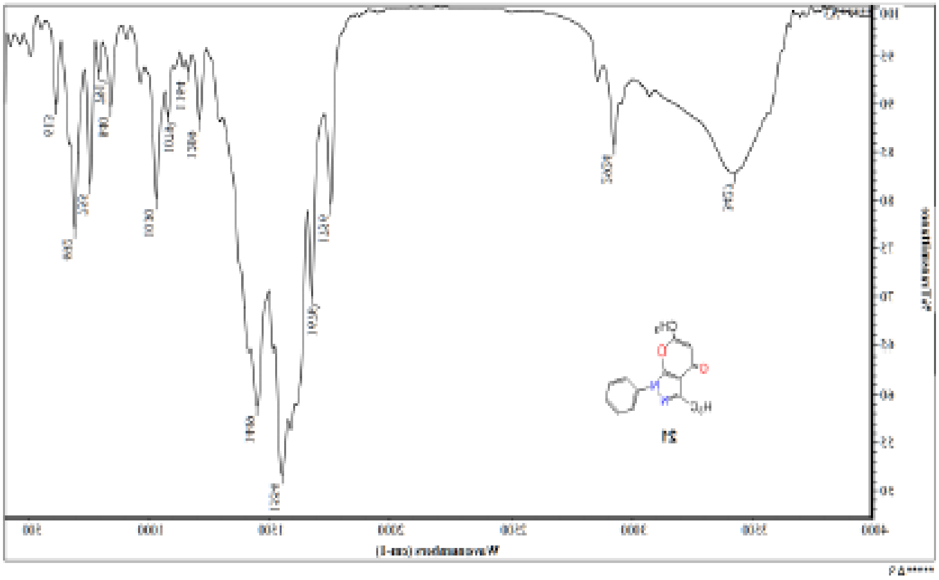

Figure S32: IR spectrum of compound **21**

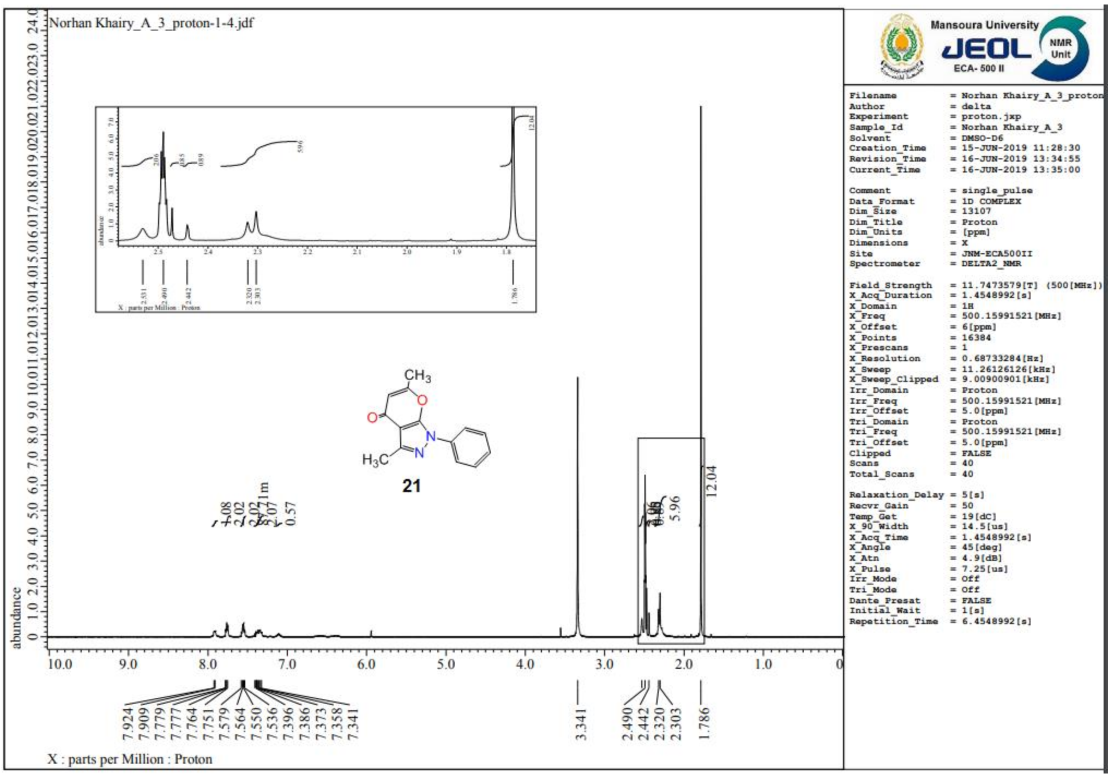

Figure S33: ^{1}H-NMR spectrum of compound **21**

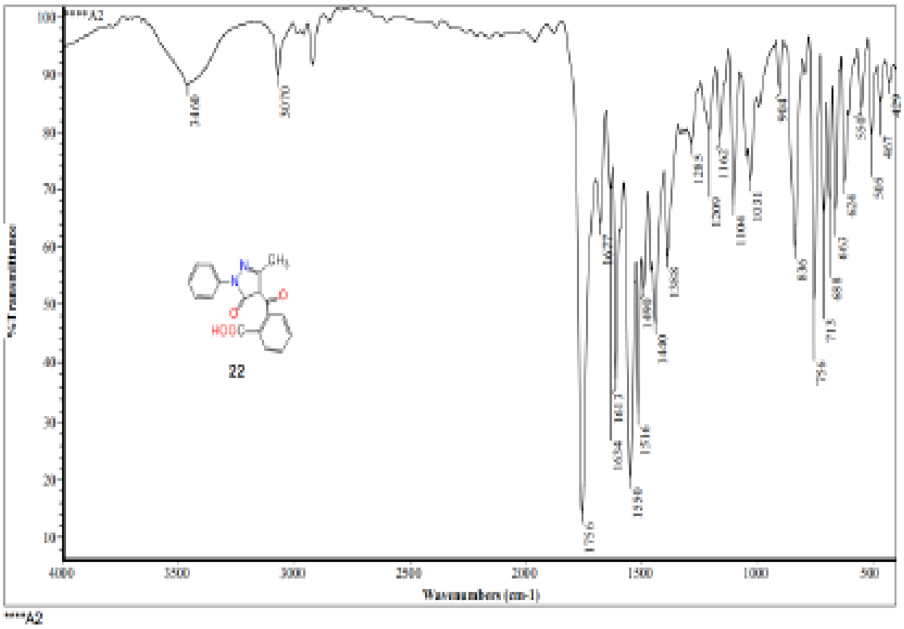

Figure S34: IR spectrum of compound **22**

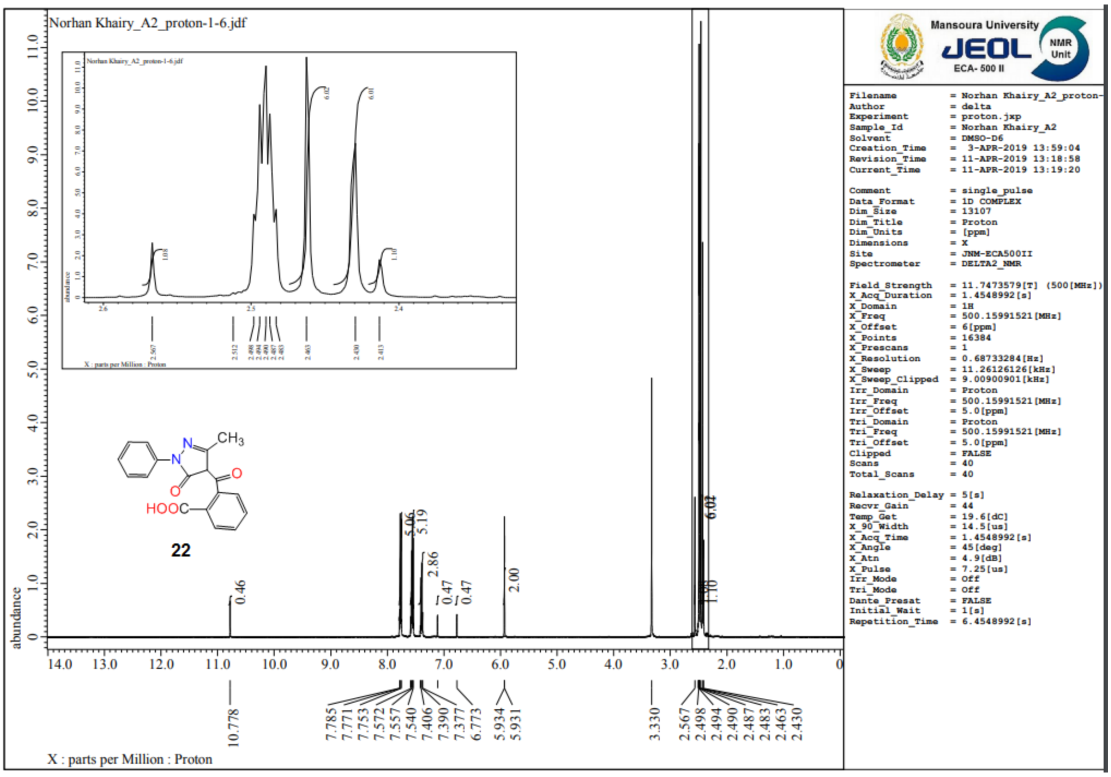

Figure S35: ^{1}H-NMR spectrum of compound **22**

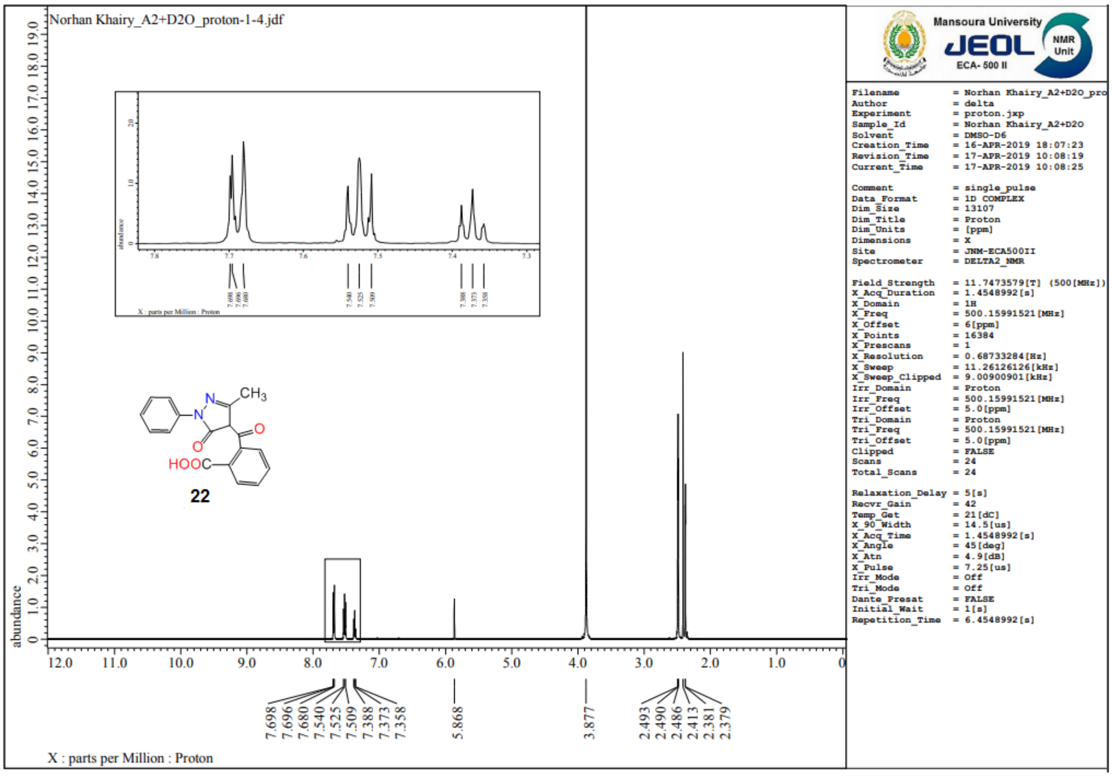

Figure S36: ^{1}H-NMR spectrum (D_2O) of compound **22**

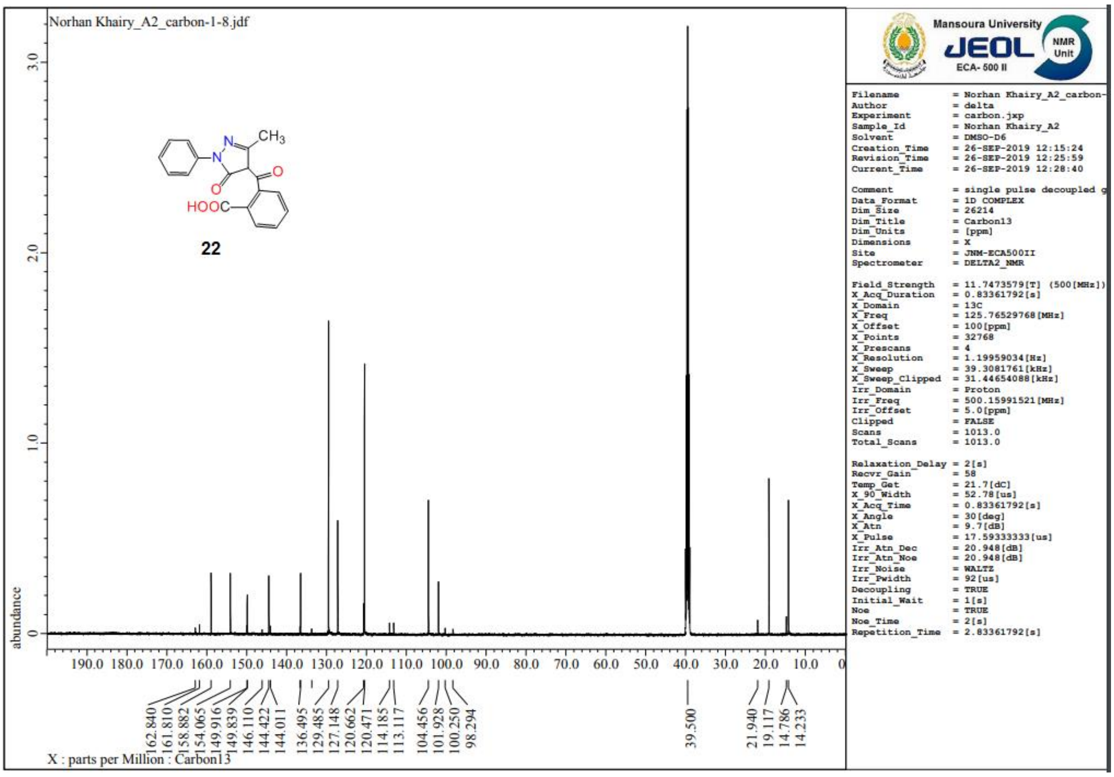

Figure S37: ^{13}C-NMR spectrum of compound **22**

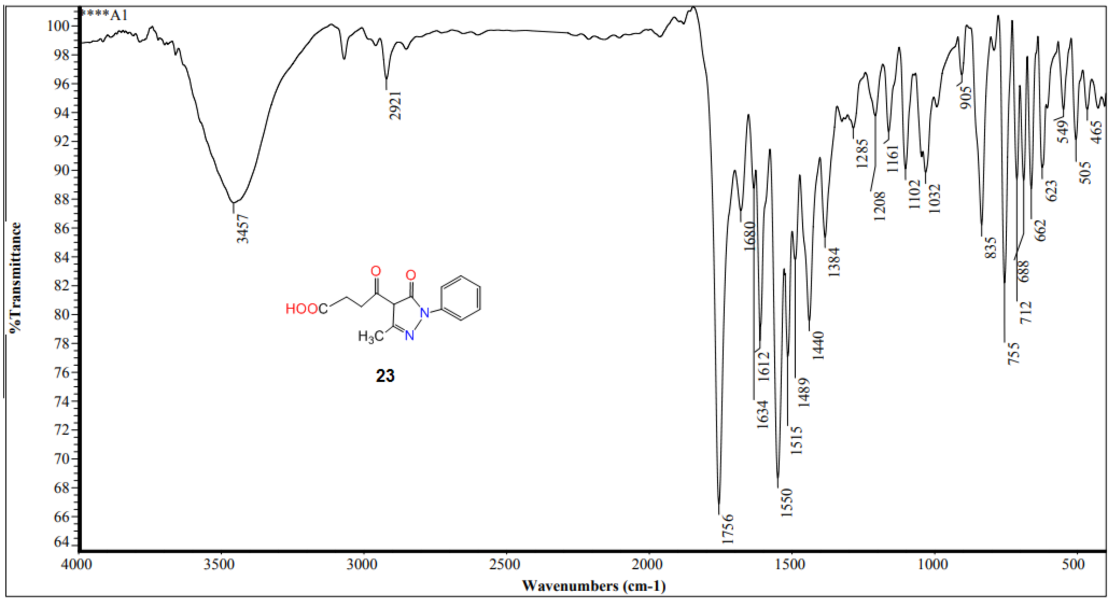

Figure S38: IR spectrum of compound **23**

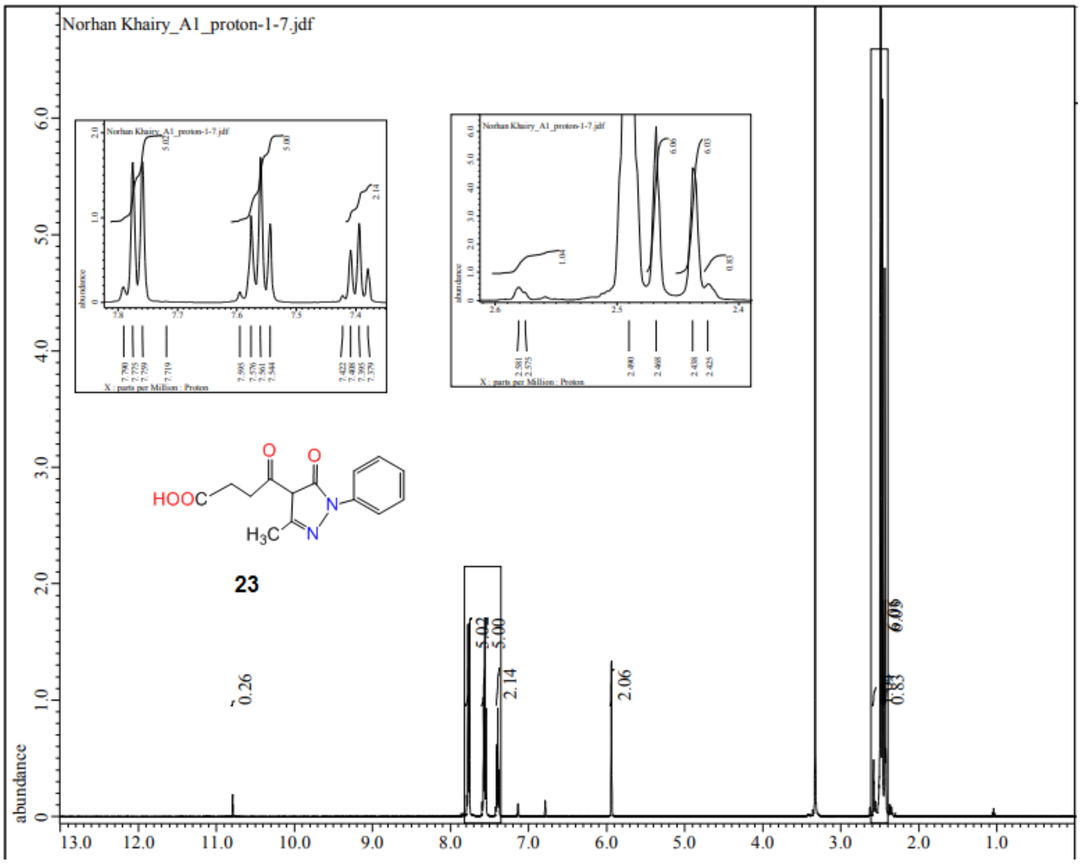

Figure S39: H-NMR spectrum of compound **23**

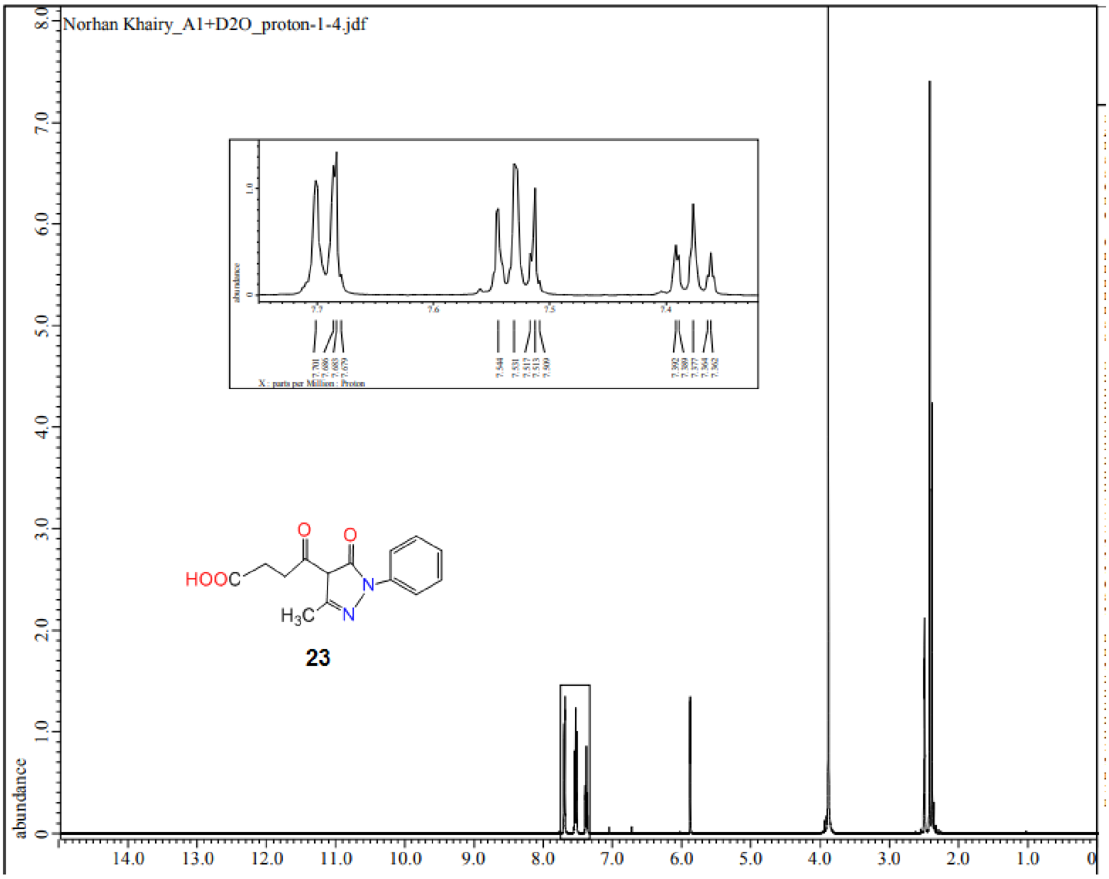

Figure S40: ^{1}H-NMR spectrum (D_2O) of compound **23**

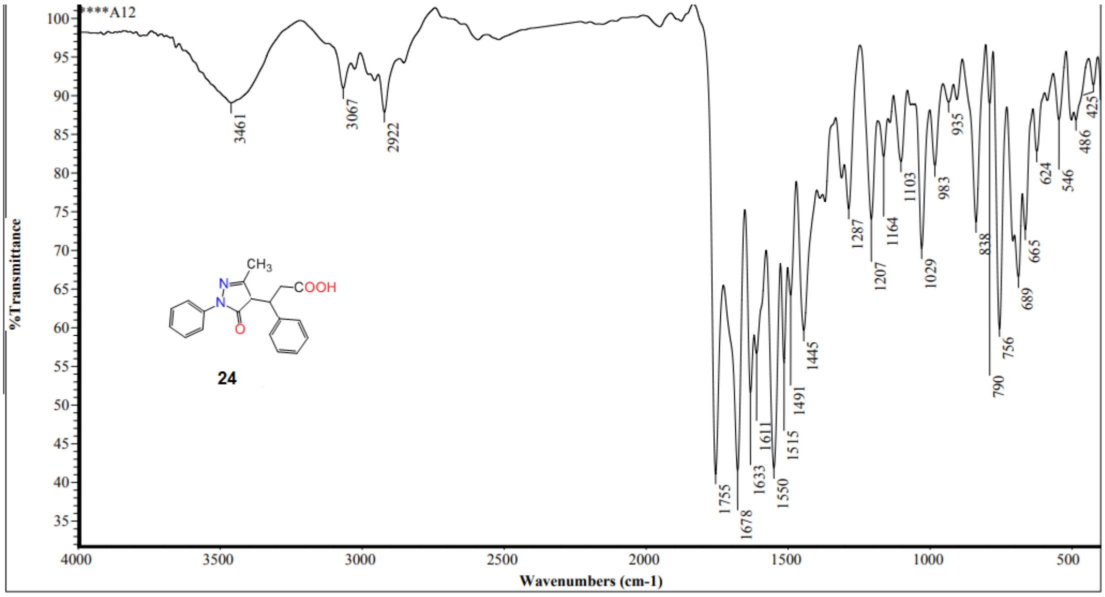

Figure S41: IR spectrum of compound **24**

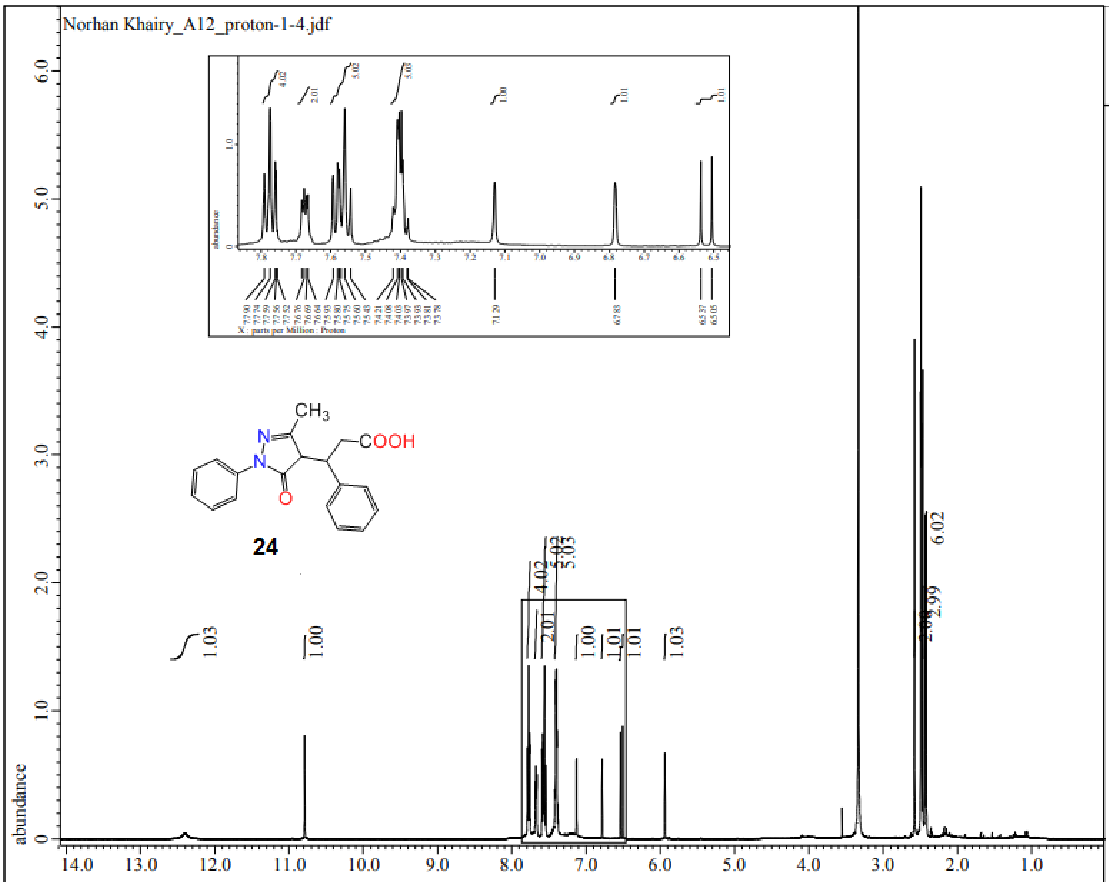

Figure S42: ^{1}H-NMR spectrum of compound **24**
